# Deficiency of copper responsive gene* stmn4* induces retinal developmental defects

**DOI:** 10.1007/s10565-024-09847-8

**Published:** 2024-01-22

**Authors:** YuanYuan Jing, Yi Luo, LingYa Li, Mugen Liu, Jing-Xia Liu

**Affiliations:** 1 College of Fisheries, Key Laboratory of Freshwater Animal Breeding, Ministry of Agriculture, Huazhong Agricultural University, Wuhan, 430070 China; 2 Key Laboratory of Molecular Biophysics of the Ministry of Education, College of Life Science and Technology, Huazhong University of Science and Technology, Wuhan, 430074 Hubei China

**Keywords:** Retinal cells, Mitosis, Cell cycle, Apoptosis, Copper, *stmn4*

## Abstract

As part of the central nervous system (CNS), the retina senses light and also conducts and processes visual impulses. The damaged development of the retina not only causes visual damage, but also leads to epilepsy, dementia and other brain diseases. Recently, we have reported that copper (Cu) overload induces retinal developmental defects and down-regulates microtubule (MT) genes during zebrafish embryogenesis, but whether the down-regulation of microtubule genes mediates Cu stress induced retinal developmental defects is still unknown. In this study, we found that microtubule gene *stmn4* exhibited obviously reduced expression in the retina of Cu overload embryos. Furthermore, *stmn4* deficiency (*stmn4*^*−/−*^) resulted in retinal defects similar to those seen in Cu overload embryos, while overexpression of *stmn4* effectively rescued retinal defects and cell apoptosis occurred in the Cu overload embryos and larvae. Meanwhile, *stmn4* deficient embryos and larvae exhibited reduced mature retinal cells, the down-regulated expression of microtubules and cell cycle-related genes, and the mitotic cell cycle arrests of the retinal cells, which subsequently tended to apoptosis independent on *p53*. The results of this study demonstrate that Cu stress might lead to retinal developmental defects via down-regulating expression of microtubule gene *stmn4*, and *stmn4* deficiency leads to impaired cell cycle and the accumulation of retinal progenitor cells (RPCs) and their subsequent apoptosis. The study provides a certain referee for copper overload in regulating the retinal development in fish.

**Graphical Abstract:**

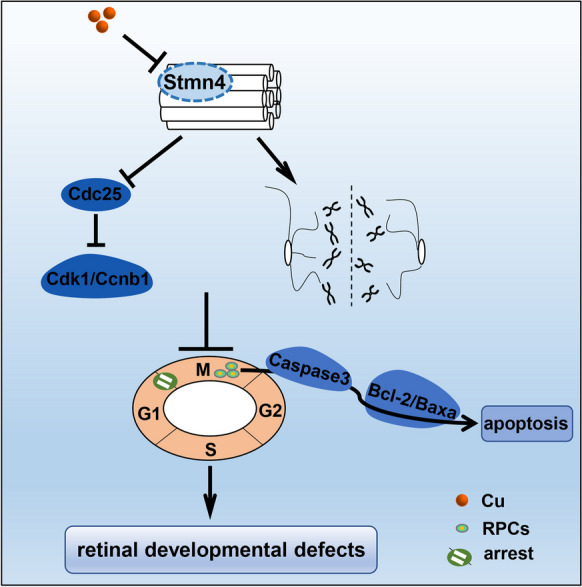

**Supplementary Information:**

The online version contains supplementary material available at 10.1007/s10565-024-09847-8.

## Background

Stathmin family proteins play their functions by promoting mitotic spindle disassembly and the subsequent exiting from mitosis (Jourdain et al. 1997). The absence of Stathmin expression leads to accumulation of cells in the G2/M phases and is associated with severe mitotic spindle abnormalities and difficulty in the exit from mitosis (Rubin and Atweh 2004), indicating Stathmins are important for microtubule (MT) dynamics (Charbaut et al. 2001) and are very crucial in the process of mitosis, cell cycle and cell differentiation (Mistry and Atweh 2002). Stathmins are involved in neuronal development, plasticity and regeneration (Chauvin and Sobel 2015), are regarded as neuronal microtubule-regulatory proteins (Burzynski et al. 2009; Levy et al. 2011; Shih et al. 2014), and play crucial roles in mitosis (Belletti and Baldassarre 2011). The overexpression or downregulation of *Stathmins* disrupts the correct completion of cell division, and they are important targets for the main regulatory factor of M-phase cyclin-dependent kinase 1 (CDK1) (Belletti and Baldassarre 2011).

Some special findings in STMN4 have been reported recently, such as STMN4 is the only response protein in the Stathmin family proteins to optic nerve (ON) axotomy in rats (Nakazawa et al. 2005) and the only one to induce the differentiation of PC12 cells in vitro (Beilharz et al. 1998), suggesting that *STMN4* may play a crucial role among the neuronal development process, especially in optical development. Meanwhile, STMN4 possesses a unique N-terminal domain, which makes the function of the full-length STMN4 not only has MT destabilization activities similar to Stathmins, but also enhances the binding affinities of STMN4 for MTs (Nakao et al. 2004). However, whether and how Stathmins, especially STMN4, act in optical or retinal development, have rarely been studied.

Recent studies have reported that Cu overload causes developmental defects of retinal cells in zebrafish embryos and larvae (Li et al. 2023; Zhao et al. 2020), and *stmn4* is significantly down-regulated in Cu overload hematopoietic stem and progenitor cells (HSPCs) (Li et al. 2023). However, whether Cu overload induces the development defects of retinal cells by down-regulating *stmn4* expression, and the potential mechanisms of *stmn4* in regulating the development of zebrafish retinal cells, remain unknown.

Optical retina is very special somatosensory tissue for sensing light, which is required in the survival behavior regulation such as foraging and avoiding natural enemies in healthy aquaculture in fish. Zebrafish, as a model organism, with advantages of in vitro fertilization, rapid development, and embryonic transparency, has been served in embryonic developmental studies for long time (Malicki et al. 2016). Studies in the optical retinal cell development, cell proliferation and differentiation are precisely coordinated for the development and growth of zebrafish eyes (Easter and Malicki 2002; Stenkamp 2015).

In this study, we found the down-regulated expression of *stmn4* in retina in Cu overload embryos and larvae, and asked whether the down-regulation of *stmn4* mediated copper overload induced developmental defects of retinal cells. Here, we unveiled that *stmn4* was required in the differentiation of retinal progenitor cells (RPCs). *Stmn4* knockout led to small eye and reduced retinal cells via affecting cell cycle progression and differentiation of RPCs and the subsequent cell apoptosis during zebrafish embryogenesis. Our findings here reveal the critical roles of *stmn4* in responding Cu overload in retinal cell development.

## Material and methods

### Maintenance of zebrafish stocks and embryo and larvae collection

The maintenance and breeding of zebrafish (*Danio rerio*) were performed as described previously (Tai et al. 2022). The following lines were used in this study. Wild-type (WT) zebrafish, *stmn4* deficient homozygous mutant (*stmn4*^*−/*−^) (Li et al. 2023), Tg(Huc:EGFP)(CZ160)(China Zebrafish Resource Center, http://www.zfish.cn/), and Tg(*stmn4*^*−/*−^; Huc:EGFP). The ages of embryos and larvae were expressed by hours post-fertilization (hpf). The Cu exposure solution was prepared as we performed recently (Zhang et al. 2018). Briefly, embryos were exposed to Cu^2+^ (CuSO4·5H_2_O) (Sigma, Cat#61,245) before sphere stage at 3.9 μM (Zhang et al. 2015).

### Behavior assays

In this study, *stmn4*^−/−^ and WT larvae at 96 hpf or 120 hpf in 48-well plates (one larva per well, at least 2–3 repeats for each group) were put into the Zebrafish behavior tracking system (ViewPoint Life Sciences, Montreal, Canada), and the larvae behaviors were recorded for 30 min after the larvae had been adapted for 10 min.

Meanwhile, in touch response assays, *stmn4*^−/−^ and WT larvae at 96 hpf or 120 hpf were placed in 48-well plates and were stimulated with toothpicks, and video images of their motor behaviors and escape responses after touch stimulation were recorded by Zebrafish behavior tracking system (ViewPoint Life Sciences, Montreal, Canada). The video was broken down by QuickTime Player software (version 10.4, Apple Inc.) to get different time points of each frame, and the time of each frame was displayed on each panel.

### Morpholino (MO) and mRNA injection

The morpholinos of *p53* was purchased from Gene Tools LLC (Philomath, Oregon, USA) and dissolved in ddH_2_O at 3 mM (stock solution). The full-length of *stmn4* was amplified with the specific primers, F primers: 5’ ATGACCTTGGCAGCATATCGAGACA 3’, R primers: 5’ CTACCGAACTGAAAAGCTACCAGAA 3’, and the *stmn4* full-length mRNA was synthesized using the Ambion MAXIscript T7 Kit (Cat#AM1344, Invitrogen, USA) as instructed by the manufacturer. In all experiments, the MOs and mRNAs were injected into one-cell stage embryos, respectively, with the MO dose of *p53* at 0.6 mM, and the concentrations of *stmn4* mRNA at 200 ng/µL.

### Real-time quantitative PCR (qRT-PCR) analysis

Zebrafish embryos at 16 hpf, 24 hpf, and 48 hpf, separately, were used for total RNA extraction. In this study, the expressions of *stmn1a*, *stmn1b*, *stmn2a*, *stmn2b*, *stmn3*, *stmn4* in *stmn4*^−/−^ and WT embryos at 24 hpf, of *p21*, *p130*, *cyclinA2*, *cdk1*, *cyclinE*, *cyclinD*, *cdc25b*, *cyclinB*, *cyclinG2*, *atm*, *cyclinA1* and *cenp* in *stmn4*^*−/*−^ and WT embryos at 16 hpf and 24 hpf, and the expressions of *p53*, *bcl2*, *caspase8*, *baxa* in the embryos at 24 hpf and 48 hpf, were tested, and qRT-PCR was conducted as we reported previously (Zhang et al. 2020). The primer sequences were listed in Table S1. Each sample was run in triplicate and repeated at least three times. Differences were calculated by the 2^−ΔΔCt^ comparative quantization method using *18s* or *gapdh* as an internal control.

### Whole-mount in situ hybridization (WISH)

WISH was performed as previously described (Zhang et al. 2020). Probes for *myelin basic protein a* (*mbp*), *proteolipid protein 1a* (*plp1a*) and *vimentin* (*vim*) were synthesized as we performed previously (Zhang et al. 2020), and probes for other genes tested in this study were synthesized using T7 in vitro transcription polymerase (Roche Molecular Biochemicals, Germany) and DIG RNA labeling kit (Roche Molecular Biochemicals, Germany), sequences for all primers used in this study were listed in Table S2. The images were captured by an optical microscope (Leica. M205FA, Germany). Data quantification and visualization were carried out using ImageJ software (NIH, Bethesda, Maryland) and GraphPad Prism 8.0, respectively. A minimum of 15 embryos per group were used for WISH analysis, and three independent experiments were performed. A representative image in each group is shown.

### Immunofluorescence and Hematoxylin–eosin (H&E) assays

Embryos and larvae at 24 hpf, 48 hpf, 72 hpf, 96 hpf and 7 dpf were fixed with 4% PFA overnight at 4 °C, and then were dehydrated with 30% sucrose PBS solution for 2 h at room temperature. Next, the permeated embryos were embedded in TissueTek® O.C.T. compound (Sakura Finetek, USA) for cryosectioning at 6 ~ 8 μm in thickness with frozen microtomy (Thermo scientific, USA). After drying at 4 °C, the sections were used for Hematoxylin and Eosin (H&E) staining, immunofluorescence assays, and in situ hybridization (ISH) assays, respectively. The H&E staining and ISH assays was performed as reported previously (Niu et al. 2014; Zhao et al. 2020). Then, high-resolution images for the H&E staining and ISH assay sections were obtained under a microscope (ZEISS Axio Imager A2) after the staining was completed.

The immunofluorescence assays were performed with the primary antibodies against Caspase3 (A0214, ABclone, 1:200), PH3 (AF3358, Affinity, 1:200), Opn1sw2 (Azb21565b, Abcepta, 1:200), Opn1lw1 (A24373, ABclone, 1:100), Rhodopsin (A7245, ABclone, 1:100), Sox2 (A0561, ABclone, 1:200) and the fluorescent secondary antibodies (AS053, ABclone, 1:500; AS058, ABclone, 1:500) at 37℃, 2 h. The cell nuclei were stained with DAPI (5 μg/ml). Immunostaining assays in frozen sections were performed as described previously (Zhao et al. 2020). The apoptosis detection was performed with a TUNNEL detection kit (Elabscience Biotechnology, Wuhan, China). The immunofluorescence and the apoptosis samples were imaged using a confocal microscope (Leica M205FA, Germany).

### BrdU labeling and EdU labeling

The *stmn4*^*−/*−^ and the control at 46 hpf and 94 hpf, respectively, were injected with BrdU (10 mM; Beyotime, Cat#ST1056) or EdU (10 mM, Abbkine, Cat#KTA2031) peritoneally, followed by incubation for 2 h and fixed in 4% paraformaldehyde (PFA). Next, the permeated embryos were embedded in TissueTek® O.C.T. compound (Sakura Finetek, USA) for cryosectioning at 6 ~ 8 μm in thickness with frozen microtomy (Thermo scientific, USA). After drying at 4 °C, the sections were used for EdU staining, or staining with BrdU Mouse mAb (1:200) antibodies according to the manufacturer’s protocol, and for other antibody immunofluorescence assays, respectively. Finally, the slices were imaged using a confocal microscope (Leica M205FA, Germany).

### Western blotting (WB)

Whole embryos and the cut head tissues at 24 hpf and 48 hpf, separately, were homogenized in Radio Immunoprecipitation Assay (RIPA) lysis buffer with proteinase inhibitor (Cat#89,900, Thermo Fisher Scientific, USA). Then, the appropriate SDS-PAGE loading buffer was separately added and the obtained samples were boiled for 10 min. Each protein sample was quantified to make sure an almost equal amount of protein in each line was separated by polyacrylamide gel electrophoresis. The separated protein was transferred to polyvinylidene fluoride (PVDF) microporous membrane (Bio-Rad Laboratories, Hercules, CA, USA). The whole embryo protein samples were used for proteins Stmn4, Sox2, Ccnb1, Cdk1, Cdc25b and α-Tubulin assays via WB, and the cut head tissues protein samples were used for proteins P53, Bcl-2 and Cleaved Caspase3 assays via WB. The blots were then blocked with 0.2% skim milk in TBS containing 0.1% Triton X-100, followed by incubation first with the primary antibodies, Stmn4 (DF4547, Affinity, 1:200), Sox2 (A0561, ABclone, 1:200), Cdc25b (A9758, ABclone, 1:200), Cyclin B1(Ccnb1) (A19037, ABclone, 1:200), Cdk1 (A11420, ABclone, 1:200), α-Tubulin (GT114, GeneTex, 1:200), P53 (80,077–1-RR, Proteintech, 1:200), Bcl-2 (A0208, ABclone, 1:200), and Caspase3 (A0214, ABclone, 1:200), respectively, and then with secondary antibody Goat anti-Rabbit lgG (H + L) in a 1:1000 dilution (Cat#BL033A, Biosharp, China). Finally, the blots were visualized using enhanced chemiluminescence (Bio-Rad Laboratories, Hercules, CA, USA). Multi Gauge V3.0 was used for quantifying the protein levels based on the band density obtained in the WB analysis.

### RNA-Sequencing (RNA-Seq) and analysis

In this study, fifty zebrafish embryos of control and *stmn4*^−/−^ mutants at 16 hpf and 24 hpf were collected separately and used for RNA extraction and RNA sequencing (RNA-Seq). RNA-Seq was performed on an Il-lumina HiSeq2000 platform by Novogene (Beijing, China). Genes with significant alterations due to *stmn4* deletion (adjusted *P* < 0.05) were defined as differentially expressed genes (DEGs). Enriched Kyoto Encyclopedia of Genes and Genomes (KEGG) pathway analysis was conducted for each sample using KOBAS v.2.0 based on the lists of DEGs. Gene Ontology (GO) analysis was conducted using the lists of DEGs by GO seq Release 2.12. Hierarchical clustering was performed by TIGR Multi Experiment Viewer (MeV) to generate different heatmaps.

### Statistical analysis

RNA extraction, protein extraction and other experimental samples were collected, about 50 embryos in each group. The sample size for different experiments in each group was larger than 10 embryos (n > 10) with 3 biological replicates for WISH test. The data were quantified by Image J and analyzed and visualized by GraphPad Prism 8.0. The results were passed by* t* test and post hoc Turkey's test in SPSS (20.0) software. The statistical significance between groups was determined at *P* < 0.05 (*), *P* < 0.01 (**) or *P* < 0.001 (***). Data are expressed as the mean ± standard deviation (SD) for normal distribution and median (range) for no-normal distribution.

## Results

### *Stmn4*^−/−^ exhibits eye developmental defects

**Fig. 1:**
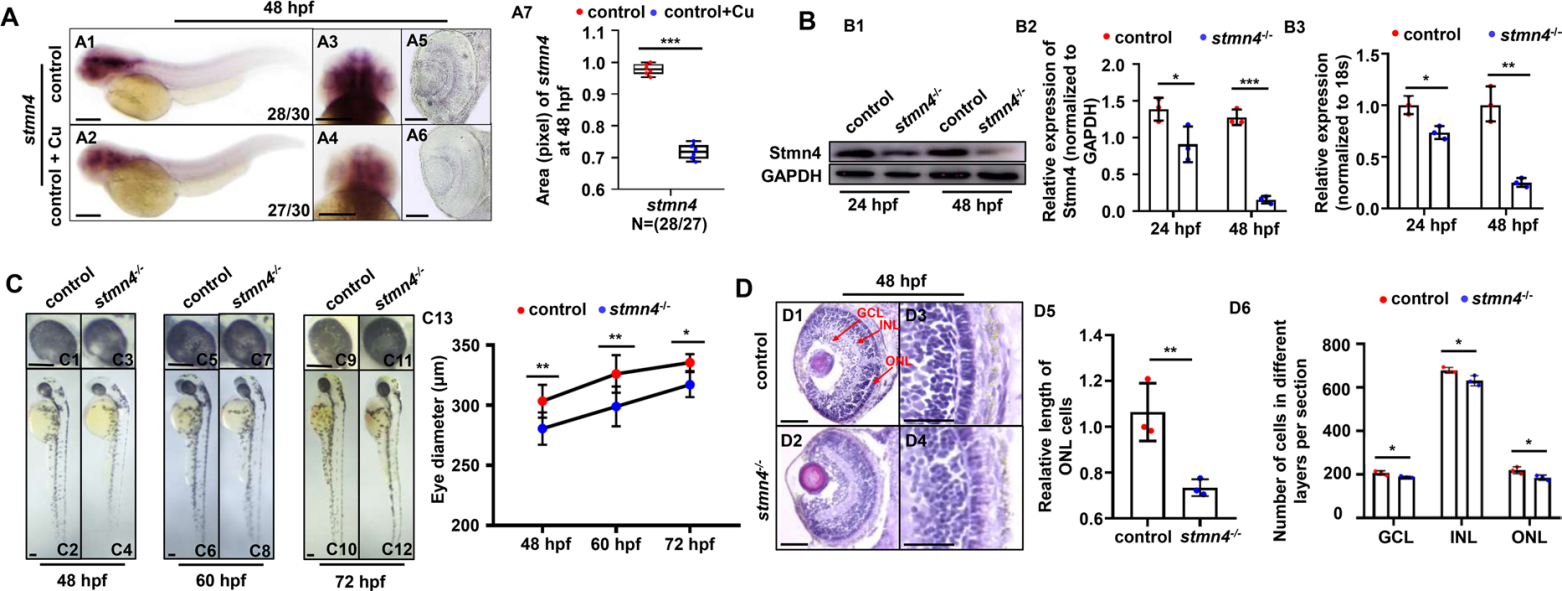
*Stmn4* deficiency led to eye morphological defects. (**A**) Copper (Cu) stress induced obviously reduced expression of *stmn4* in eyes during zebrafish embryogenesis (A1 − A4), cross-section of the eye (A5, A6) and the calculation of the relative expression levels of *stmn4* (A7). (**B**) Western blot detection of Stmn4 protein levels in WT and *stmn4*^*−/−*^ at 24 hpf and 48 hpf (B1 − B2), and the mRNA levels of *stmn4* in WT and *stmn4*^*−/−*^ at 24 and 48 hpf (B3), respectively. (**C**) Embryonic and eye development in *stmn4*^*−/−*^ and WT embryos and larvae at different developmental stages (C1 − C12), and the quantification of the eye size in WT and *stmn4*^−/−^ embryos and larvae (C13), respectively. (**D**) HE staining showed the difference in the eyes of WT and *stmn4*^−/−^embryos at 48 hpf (D1 − D4), and the calculation data (D5, D6). ONL, outer nuclear layer; INL, inner nuclear layer; GCL, ganglion cell layer. A1, A2, lateral view, anterior to the left, and dorsal to the up; A3, A4, head to the up, and dorsal to the down; C1 − C12, lateral view, anterior to the up, and dorsal to the right. Scale bar, 200 μm (A1 − A4), 100 μm (A5, A6, C1 − C12, D1, D2), 50 μm (D3, D4). ***P < 0.001, **P < 0.01, *P < 0.05

We have recently unveiled that the microtubule related DEGs were significantly enriched in the GO terms, in both Cu overload zebrafish embryos and their HSPCs at 24 hpf and 33 hpf, in which *stmn4* was significantly down-regulated (Li et al. 2023). Meanwhile, Cu overload zebrafish embryos and larvae exhibit dysfunctional locomotor behavior, microphthalmia, and retinal developmental defects (Zhao et al. 2020), and *stathmins* have been reported to be required in neural cell development (Beilharz et al. 1998; Levy et al. 2011; Zhao et al. 2020). Thus, in this study, we asked whether the down-regulation of *stmn4* mediated the retinal developmental defects occurred in Cu overload zebrafish embryos. The expression of *stmn4* was significantly down-regulated in retina in Cu overload zebrafish embryos (Figs. 1A1 − A6), and *stmn4* transcripts were predominantly distributed in the head of zebrafish embryos and larvae (Figs. S1A1 − A16). *Stmn4* knockout zebrafish line with an 8 bp deletion in exon2 (*stmn4*^*−/−*^) has been constructed and reported recently (Li et al. 2023). In this study, we unveiled the obviously decrease in protein and the mRNA levels of Stmn4 in *stmn4*^*−/−*^ embryos and larvae (Fig. 1B1 − B3). Meanwhile, the mRNA transcripts of *stathmin* family genes (*stmn1a, stmn1b, stmn2a, stmn2b, stmn3*) were tested further, and only *stmn2b* exhibited up-regulated expression in the mutants while the others were not changed (Fig. S1B)*.* Compared with their siblings, *stmn4*^*−/−*^ mutants showed almost identical morphology but with smaller eyes (Figs. 1C1 − C13), and cells in outer nuclear layer (ONL), inner nuclear layer (INL), and ganglion cell layer (GCL), were obviously reduced in *stmn4*^*−/−*^ mutants at 48 hpf (Figs. 1D1 − D6), and at 72 hpf and 7 dpf (Figs. S1C1 − C11). Meanwhile, *stmn4*^*−/−*^ larvae generally responded more slowly to touch responses compared with their WT siblings (Figs. S1D1 − D8), and exhibited almost no differences from their WT sibling in the average acceleration and the total motion distance in behavior assays (Figs. S1E1 − E2).

### Impaired differentiation of neural cells occurs in *stmn4*^*−/−*^ embryos and larvae

**Fig. 2:**
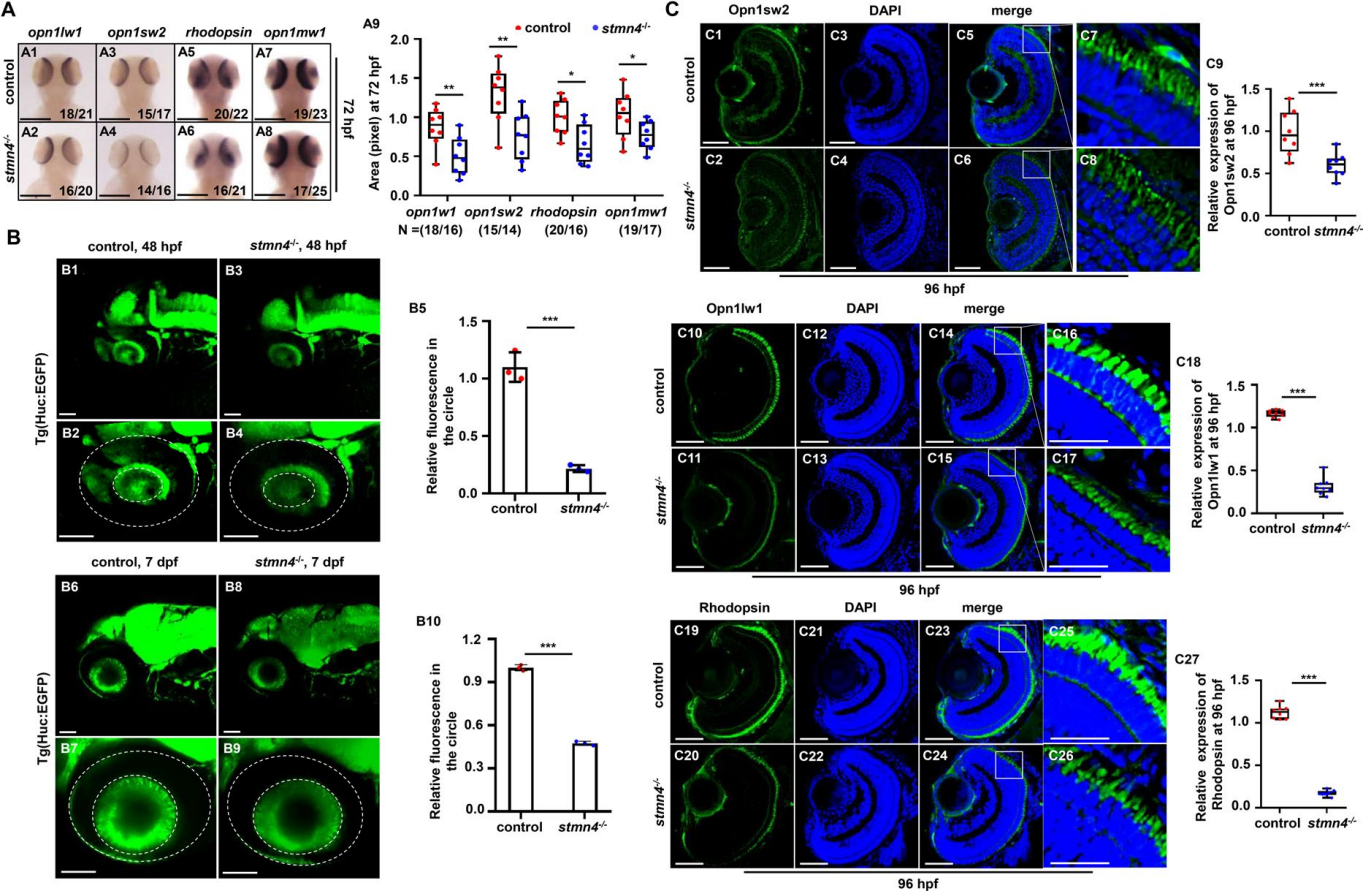
Defects of retinal cell development in *stmn4*^−/−^. (**A**) Expressions of retinal o*pn1lw1*, *opn1sw2*, *rhodopsin*, and *opn1mw1* at 72 hpf in WT and *stmn4*^−/−^ zebrafish larvae (A1 − A8), and the calculation of the relative expression levels in each sample (A9). (**B**) Expression of retinal fluorescence in Tg (Huc: EGFP) and Tg (*stmn4*^−/−^; Huc: EGFP) at 48 hpf zebrafish embryos (B1 − B4) and 7 dpf zebrafish larvae (B6 − B9), and the quantification of relative fluorescence in each sample (B5, B10). (**C**) Immunofluorescence assays for the Opn1sw2, Opn1lw1 and Rhodopsin expression at 96 hpf in WT and *stmn4*^−/−^ zebrafish larvae (C1 − C8, C10 − C17, C19 − C26), and the quantification of relative fluorescence in each sample (C9, C18, C27). A1 − A8, head to the up, and dorsal to the down; B1 − B4, B6 − B9, lateral view, anterior to the left, and dorsal to the up. Scale bar, 200 μm (A1 − A8), 100 μm (B1 − B4, B6 − B9), 50 μm (C1 − C8, C10 − C17, C19 − C26), 25 μm (C7, C8, C16 − C17, C25 − C26). ***P < 0.001, **P < 0.01, *P < 0.05

*Stmn4*^*−/−*^ mutants exhibited smaller eyes and impaired touch responses, almost phenocopied the retinal (Zhao et al. 2020) and central nervous system (CNS) (Zhang et al. 2020) developmental defects observed in Cu overload embryos and larvae. Meanwhile, the nervous system is essential for normal locomotor behavior in embryos and larvae (Granato et al. 1996), thus, we tested neurogenesis such as retinal cell development and neural cell development in the mutants subsequently. *Opn1lw1*, *opn1sw2* and *opn1mw1* (cone opsin markers), and *rhodopsin* (rod opsin marker), exhibited obviously reduced expression in retina in *stmn4*^*−/−*^ mutants (Figs. 2A1 − A9), as we reported in Cu overload embryo and larvae (Zhao et al. 2020). Huc:EGFP^+^ cells were notably reduced in *stmn4*^*−/−*^ retina at 48 hpf (Figs. 2B1 − B5) and 7 dpf (Figs. 2B6 − B10). Besides, positive immunofluorescence staining for Opn1sw2 (Figs. 2C1 − C9) and Opn1lw1 (Fig. 2C10 − C18) showing cone cells, respectively, and for Rhodopsin (Figs. 2C19 − C27) showing rod cells, were less and more disordered in *stmn4*^*−/−*^ retina. The observations here strongly demonstrated that *stmn4* deficiency induced retinal cell developmental defects, similar as we observed in Cu overload embryos and larvae recently (Zhao et al. 2020).

**Fig. 3:**
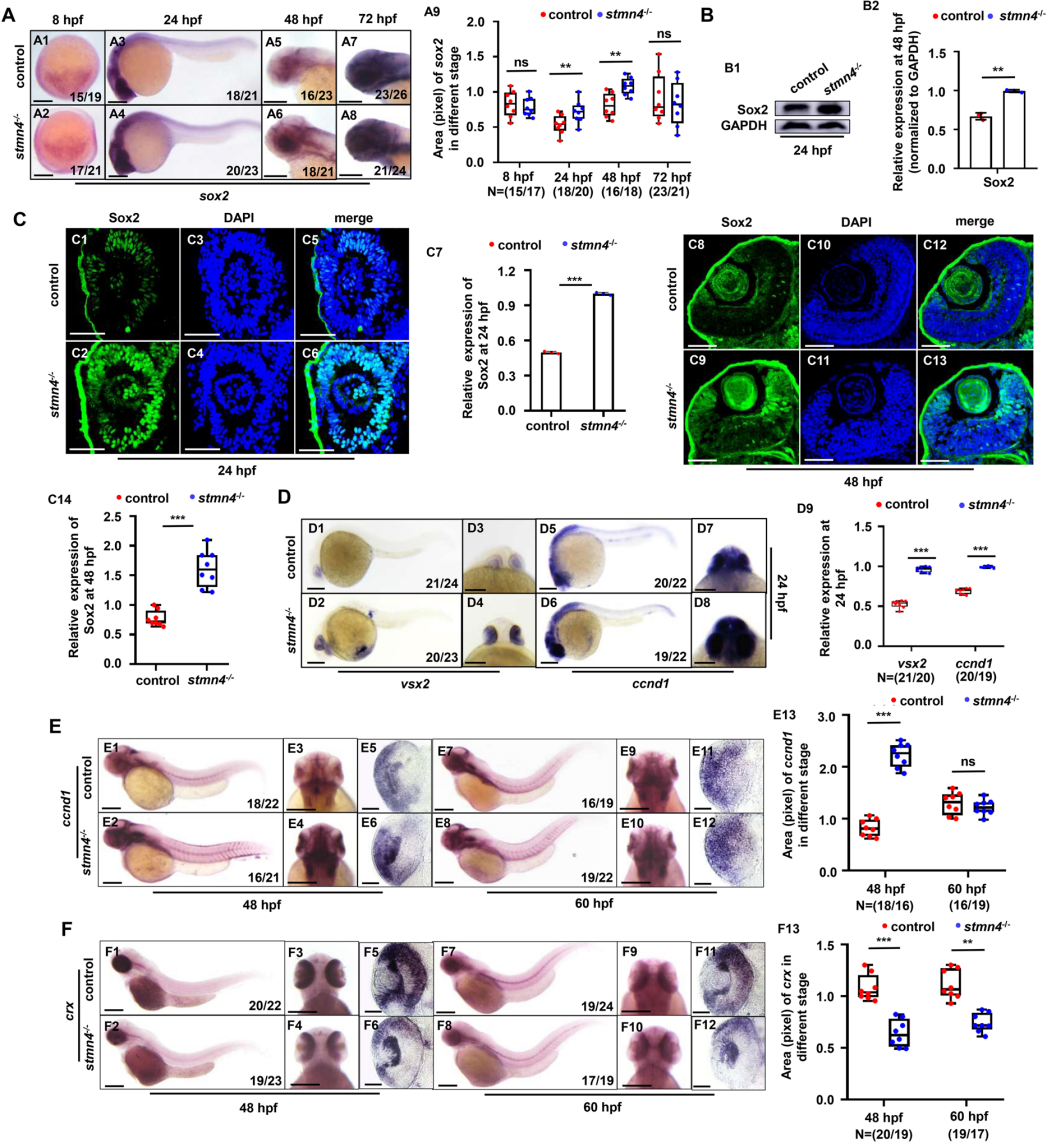
*Stmn4* deficiency induced impaired differentiation of RPCs in retina. (**A**) Expression of *sox2* at 8 hpf, 24 hpf, 48 hpf, and 72 hpf in WT and *stmn4*^−/−^ embryos and larvae (A1 − A8), and the calculation of the relative expression levels of *sox2* (A9). (**B**) Western blot analysis of Sox2 at 24 hpf in WT and *stmn4*^−/−^ embryos (B1), and the calculation of the relative expression levels of Sox2 (B2). (**C**) Immunofluorescence assays for the expression of Sox2 at 24 hpf and 48 hpf in WT and *stmn4*^−/−^ embryos (C1 − C6, C8 − C13), and the calculation of the relative expression levels (C7, C14). (**D**) Expression of *vsx2* and *ccnd1* at 24 hpf in zebrafish embryos (D1 − D8), and the calculation of the relative expression levels (D9). (**E**) Expression of *ccnd1* at 48 hpf and 60 hpf in zebrafish embryos (E1 − E4, E7 − E10), cross-section of the eye (E5, E6, E11 − E12), and the calculation of the relative expression levels (E13). (**E**) Expression of *crx* at 48 hpf and 60 hpf in zebrafish embryos (F1 − F4, F7 − F10), cross-section of the eye (F5, F6, F11 − F12), and the calculation of the relative expression levels (F13). A1 − A8, D1, D2, D5, D6, E1, E2, E7, E8, F1, F2, F7, F8, lateral view, anterior to the left, and dorsal to the up; D3, D4, D7, D8, E3, E4, E9, E10, F3, F4, F9, F10, head to the up, and dorsal to the down. Scale bar, 200μm (A1 − A8, D1 − D8, E1 − E4, E7 − E10, F1 − F4 and E7 − E10), 100 μm (E5, E6, E11 − E12, F5, F6 and F11 − F12), 50 μm (C1 − C6 and C8 − C13). ***P < 0.001, **P < 0.01, ns, not significant

Next, this study further examined the development of other neural cells in *stmn4*^*−/−*^ embryos and larvae at different developmental stages. Gene marker labelling neural progenitor and stem cells, *sox2*, was significantly increased in the whole mutants at 24 hpf, 48 hpf, and 72 hpf (Fig. 3A − C, S2A) and in *stmn4*^*−/−*^ retina (Fig. 3C, S2A). While the expression of marker genes labelling the mature neurons derived from neural precursors, *elavl3*, *rbfox3a*, *otx2b*, all exhibited reduced expression in the mutants (Figs. S2B − D). Meanwhile, gene markers *slc1a3a* for astrocyte precursors and *sox10* for oligodendrocyte precursors, also exhibited increased expression in the mutants (Fig. S3A, S4A), while the expression of genes labelling the mature glial cells, *gfap*, *vimentin*, *plp1a*, *mbp*, was significantly down-regulated in the mutants (Figs. S3B, S4B − C), suggesting generally impaired differentiation of neural cells occurred in *stmn4*^*−/−*^ embryos and larvae. The observations here were similar as the report that Cu overload larvae exhibit CNS developmental defects (Zhang et al. 2020), further suggesting the down-regulated expression of *stmn4* might mediate Cu overload induced retinal and neural system developmental defects in zebrafish embryos and larvae. In this study, we focused on the impaired differentiation of retinal cells in the *stmn4*^−/−^ mutants.

The lamination of retina is initiated by migration of neurons through mitosis to the different cell layers, where they become mature neurons and form synapses to make a link between the various cell layers (Amini et al. 2017), and the all differentiated neurons are derived from the neural precursors. Retinal stem and progenitor cells (RSPCs) distribute at the most periphery of retina, where it was considered to be a stem-cell niche (Cerveny et al. 2010; Pujic et al. 2006). Sox2, marking RSPCs, exhibited obvious increase in the mRNA (Fig. 3A1 − A9) and protein levels (Fig. 3B1, B2) in the whole mutants, and at the most periphery of retina (Fig. 3C1 − C14), suggesting abnormal accumulation of RSPCs in *stmn4*^*−/−*^ retina. Meanwhile, the signals of retinal progenitor cell (RPC) markers *vsx2* and *ccnd1* were obviously up-regulated in *stmn4*^*−/−*^ at 24 hpf (Fig. 3D1 − D9), 48 hpf and 60 hpf (Fig. 3E, S5A), but the signals of neuronal marker *crx* were reduced in *stmn4*^*−/−*^ at 48 hpf and 60 hpf (Fig. 3F), and the signals of mature neuron makers *elavl3* and *rbfox3a* were also reduced in *stmn4*^*−/−*^ mutants at 48 hpf (Figs. S2B − C), suggesting *stmn4* deficiency damaged the differentiation process of *crx* labelling neurons derived from *vsx2* and *ccnb1* labelling RPCs in retina and of the following.

**Fig. 4:**
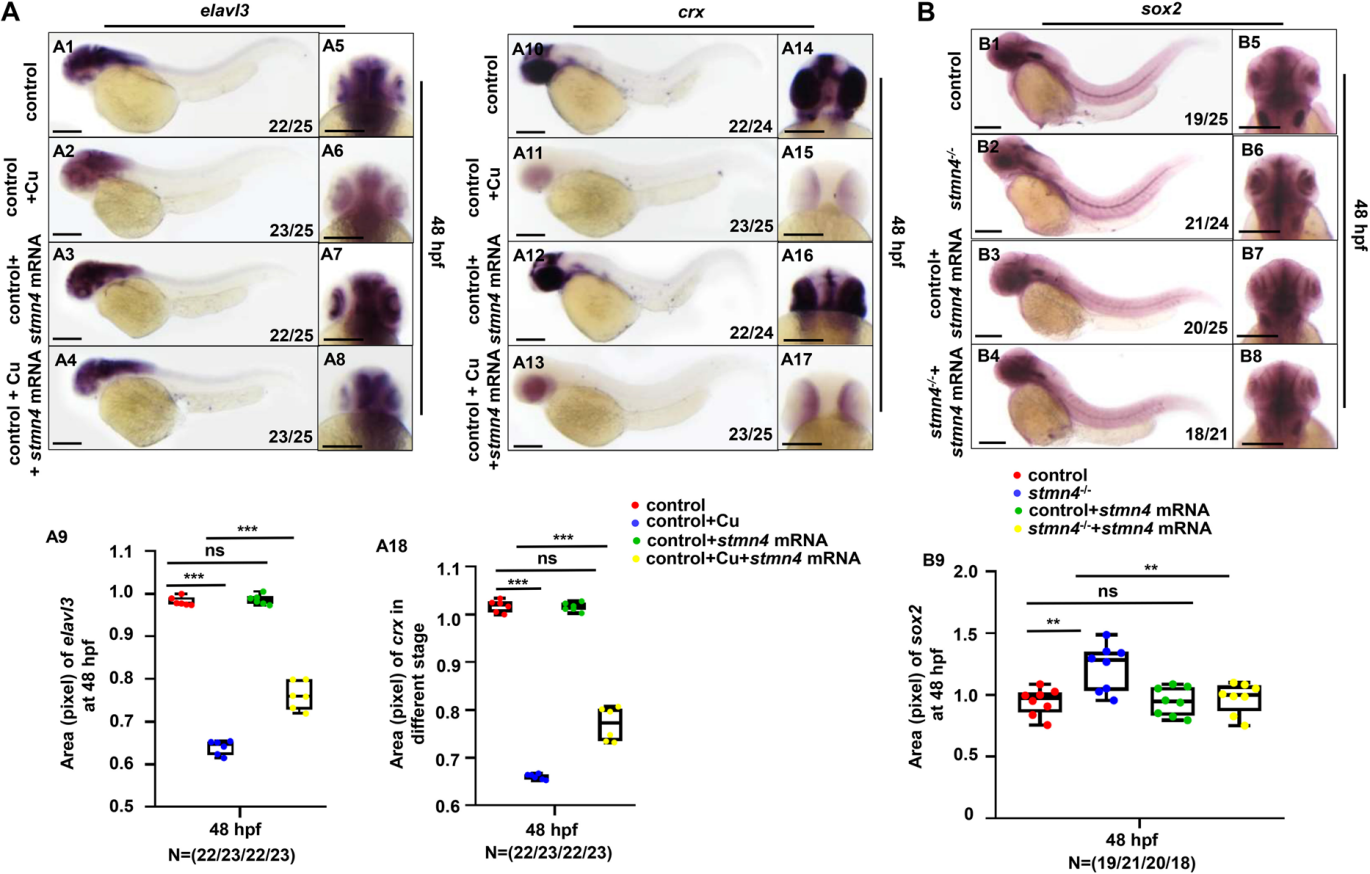
*Stmn4* mRNA could effectively rescue retinal developmental defects in both Cu stressed embryos and *stmn4*−/− mutants. (**A**) *Stmn4* mRNA effectively rescued the changed expression of *elavl3* and *crx* in retina in Cu stressed embryos (A1 − A8, A10 − A17), and the calculations of the relative expressions of *elavl3* and *crx* in embryos from each group (A9, A18). (**B**) *Stmn4* mRNA effectively rescued the changed expression of *sox2* in *stmn4*^−/−^ mutants (B1 − B8), and the calculations of the relative expressions of *sox2* in embryos from each group (B9). A1 − A4, A10 − A13, B1 − B4, lateral view, anterior to the left, and dorsal to the up; A5 − A8, A14 − A17, B5 − B8, head to the up, and dorsal to the down. Scale bar, 200 μm (A1 − A8, A10 − A17, B1 − B8). ***P < 0.001, **P < 0.01, *P < 0.05, ns, not significant

The obviously reduced expressions of mature neuron marker *elavl3* (Fig. 4A1 − A4) and neuronal marker *crx* (Fig. 4A10 − A13) while obviously increased expression of RPC marker *ccnd1* (Figs. S5C1 − C4) were also observed in retina in Cu overload embryos and larvae. On the contrary, overexpression of *stmn4* mRNA could obviously increase the expressions of the retinal markers *opn1mw1*, *rhodopsin*, and *opn1sw2* (Figs. S5B) in WT zebrafish embryos and larvae. Meanwhile, overexpression of *stmn4* mRNA could not only effectively rescue the increased expression of *sox2* to nearly normal level in retina in *stmn4*^*−/−*^ embryos (Fig. 4B1 − B9), effectively rescue the reduced expression of retinal genes *opn1mw1*, *opn1sw2*, and *rhodopsin* in the mutants (Figs. S5B), but also effectively rescue the increased expression of *ccnb1* and the reduced expression of *elavl3*, while only slightly rescue the expression of *crx* in Cu overload embryos and larvae (Fig. 4A1 − A18, S5C). Additionally, overexpression of *stmn4* mRNA could also effectively rescue the increased retinal cell apoptosis in Cu overload embryos (Fig. S6). Taken all of the aforementioned results together, with the reports that Cu overload induces retinal rod and cone cell developmental defects via stress induced cell apoptosis (Zhao et al. 2020), we speculated that the down-regulated expression of *stmn4* might be another potential contributor to Cu overload induced retinal developmental defects and cell apoptosis.

### M-Phase arrest in retinal cells in *stmn4*^*−/−*^

**Fig. 5:**
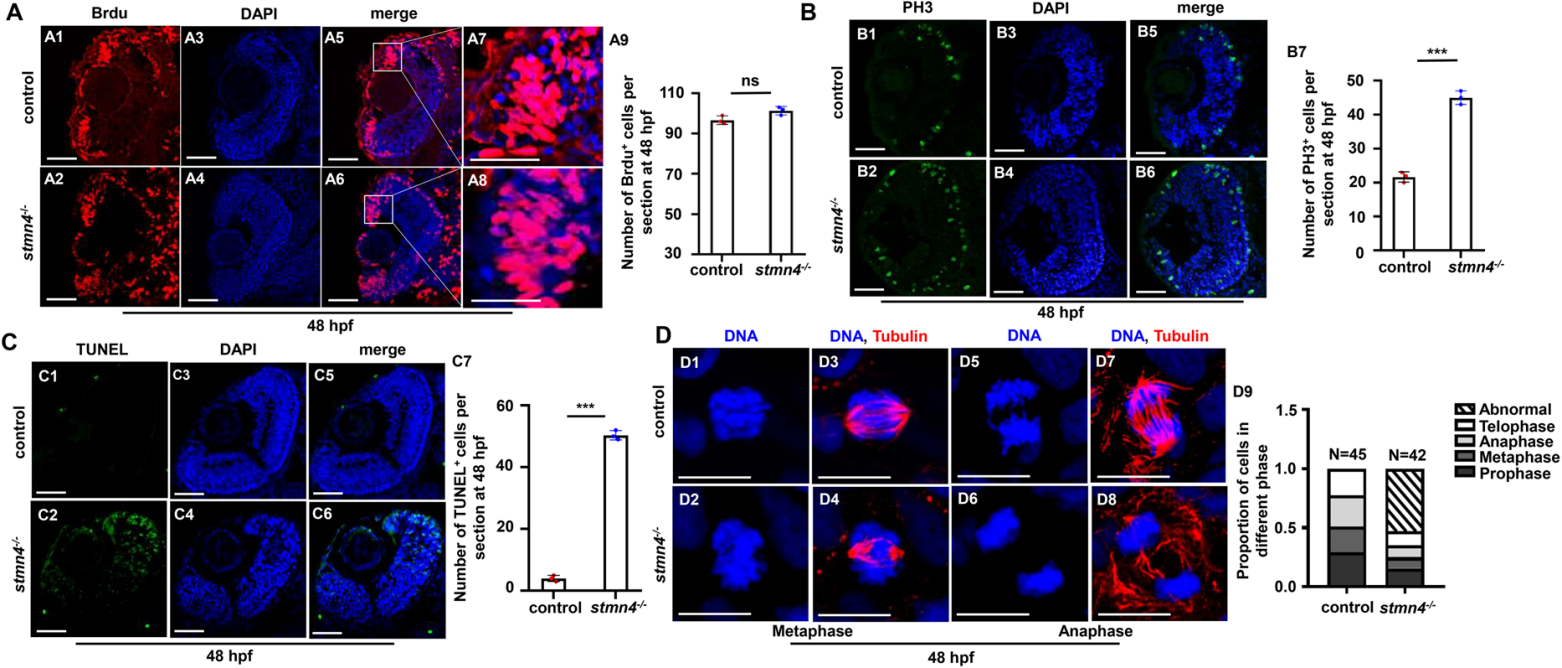
RPCs in *stmn4*^−/−^ mutants arrested in M-phase and showed abnormal mitosis. (**A**) Brdu cell proliferation assays in WT and *stmn4*^−/−^ zebrafish embryos at 48 hpf (A1 − A8) and the calculation data (A9). (**B**) PH3 cell proliferation assays in WT and *stmn4*^−/−^ zebrafish embryos at 48 hpf (B1 − B6) and the calculation data (B7). (**C**) TUNEL assays in WT and *stmn4*^−/−^ zebrafish embryos at 48 hpf (C1 − C6) and the calculation data (C7). (**D**) Immunofluorescence assays for cytoskeleton Tubulin in zebrafish embryo at 48 hpf (D1 − D8) and the calculation data (D9). Scale bar, 50 μm (A1 − A6, B1 − B6, C1 − C6), 25 μm (A7, A8), 10 μm (D1 − D8). ***P < 0.01, ns, not significant

The accumulation of RPCs occurring in *stmn4*^*−/−*^ retina suggests that the RPCs are either hyper-proliferative or arrested in the cell cycle, thus, we investigated the cell cycle progression in retina with BrdU incorporation as the S-phase marker and phosphorylated histone H3 (PH3) as the M-phase marker. It is known that the cell cycle length of retinal cells in the early stages of development is approximately 6 to 8 h (Li et al. 2000; Wehman et al. 2007), in this study, we chose zebrafish embryos at 48 hpf for cell cycle analysis in retina. There was no significant change in the BrdU^+^ signals in retina between WT and *stmn4*^*−/−*^ embryos at 48hpf (Fig. 5A1 − A9), but PH3^+^ cells showed sensibly increased in *stmn4*^*−/−*^ retina, implying that RPCs were more likely to be blocked during the M-phase (Fig. 5B1 − B7). Meanwhile, the increase of abnormal microtubule signals of *stmn4*^*−/−*^ retinal cells were observed, either in metaphase or in anaphase, and the microtubules during metaphase and anaphase were scattered and disorganized (Figs. 5D1 − D9). Additionally, the increase of cell apoptosis occurred in the most periphery of retina in *stmn4*^*−/−*^ (Fig. 5C1 − C7). These results suggested normal function of *stmn4* was required for RPCs in cell cycle, and *stmn4* deficiency increased cell death in the most periphery of retina (Fig. 5C1-C7) where might be Sox^2+^ cells (Fig. 3C8-C14).

**Fig. 6:**
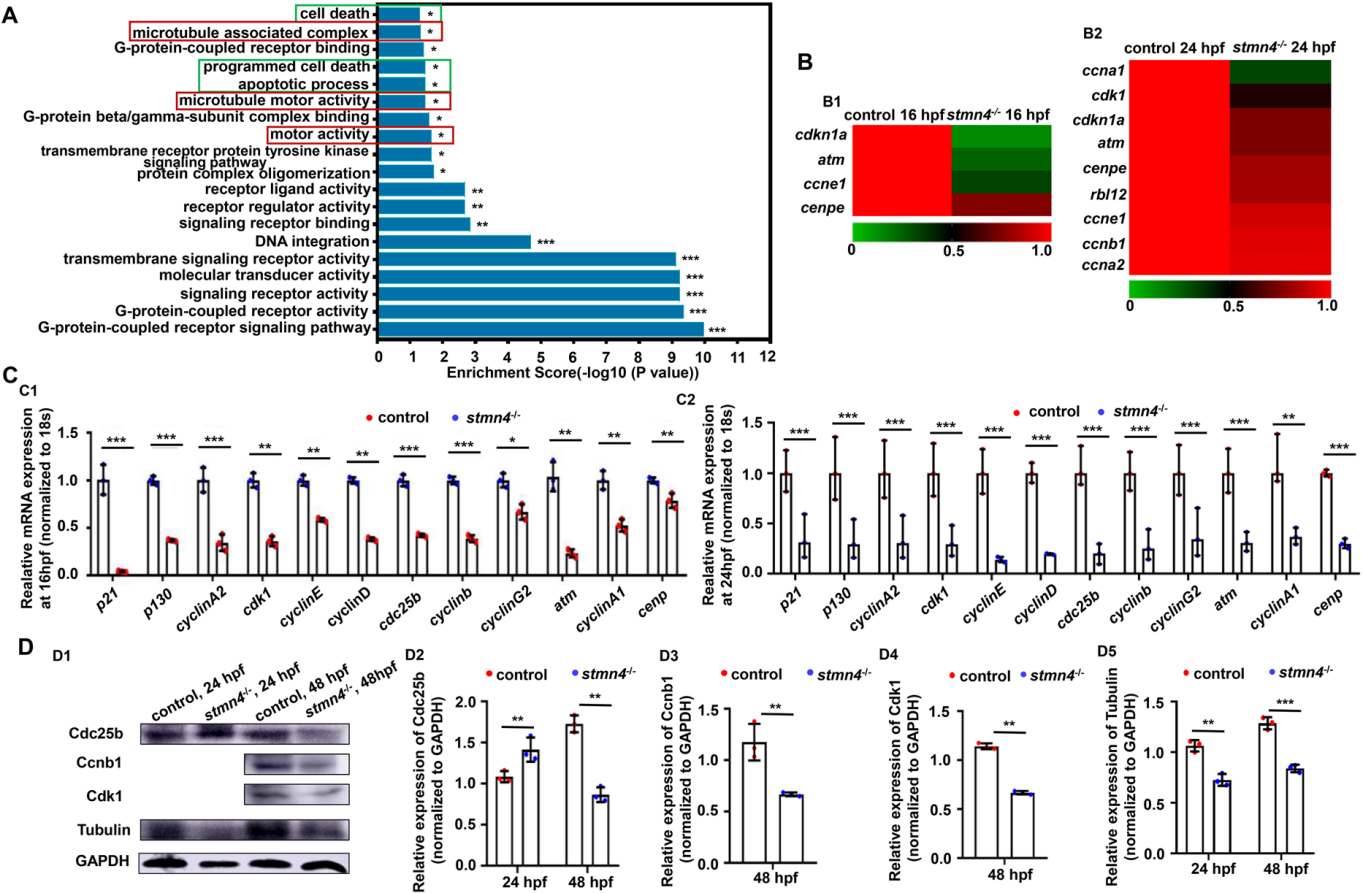
Expression of cell cycle genes in *stmn4*^***−/−***^ mutants. (**A**) GO pathway of apoptosis and mitosis were enriched for DEGs in *stmn4*^*−/−*^ mutants at 24 hpf. (**B**) Heatmaps of the down-regulated cell cycle related DEGs (B1, B2). (**C**) qRT-PCR assays for the cell cycle related genes in zebrafish embryo at 16 hpf and 24 hpf (C1, C2). (**D**) Expression of Cdc25b, Ccnb1, Cdk1, Tubulin in zebrafish embryo at 24 hpf and 48 hpf (D1), and the calculation of the protein levels in each sample (D2 − D5). ***P < 0.001, **P < 0.01, *P < 0.05

We next wondered whether the expressions of cell cycle-related regulators have also been affected with *stmn4* deficiency. Transcriptome in *stmn4*^*−/−*^ embryos were tested and the data showed that GO terms related to apoptosis (green box) and mitosis (red box) were enriched for DEGs (Fig. 6A), and cell cycle-related regulators exhibited differential expressions and were down-regulated in *stmn4*^*−/−*^ embryos (Fig. 6B1 − B2), which were verified further by qRT-PCR assays and were significantly down-regulated at both 16 hpf and 24 hpf (Fig. 6C1 − C2). The protein levels of cell cycle-related key regulators (Cdc25b, Cdk1, Ccnb1 and Tubulin) (Wang 2022) were tested further in *stmn4*^*−/−*^ mutants, and their protein levels were all reduced in the whole *stmn4*^*−/−*^ embryos at 48 hpf (Fig. 6D1 − D5). Particularly, Tubulin protein was reduced more significantly at 24 hpf in the mutants (Fig. 6D1, D5). These results indicated that *stmn4* could also indirectly regulate cell cycle processes by regulating expressions of cell cycle-related proteins.

### Apoptosis occurs in retinal cells in *stmn4*^*−/−*^

**Fig. 7:**
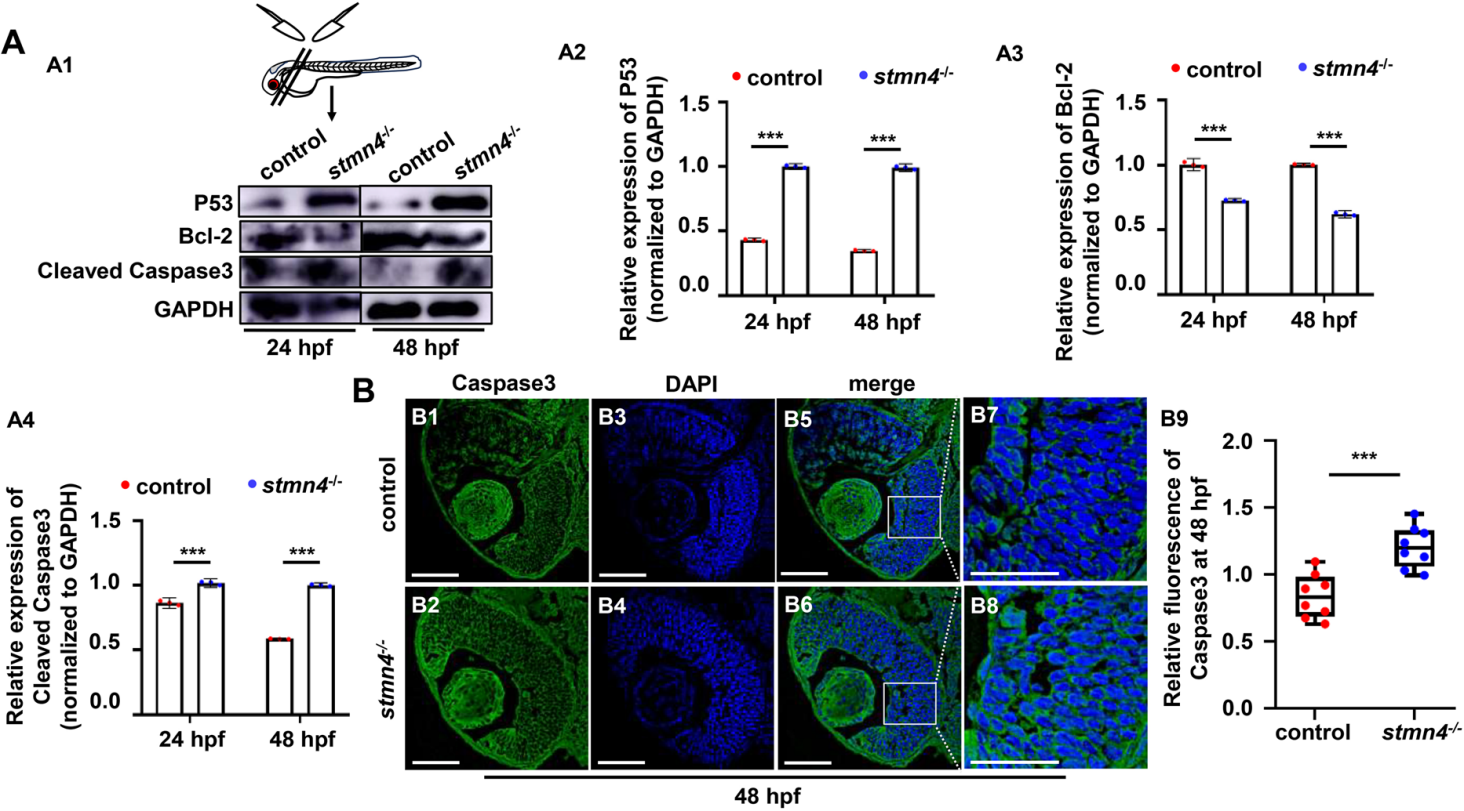
*Stmn4* deficiency led to changed expressions of apoptotic proteins. (**A**) Expression of P53, Bcl-2, and Cleaved Caspase3 in head of WT and *stmn4*^−/−^ mutants at 24 hpf and 48 hpf (A1), respectively, and the calculation of the protein levels in each sample (A2 − A4). (**B**) Immunofluorescence assays of Caspase3 in WT and *stmn4*^−/−^ retina at 48 hpf (B1 − B8) and the calculation data (B9). Scale bar, 50 μm (B1 − B6), 25 μm (B7, B8). ***P < 0.001

TUNEL assays unveiled obviously cell apoptosis occurred in retina in *stmn4*^*−/−*^ mutants (Fig. 5C1 − C7), consistently, GO terms of cell apoptosis, cell death, apoptotic process, and etc., were significantly enriched for the DEGs in *stmn4*^*−/−*^ mutants (Fig. 6A, S7A1). Meanwhile, genes in apoptotic process, such as *bcl-2*, *caspase10*, *baxa*, and etc., exhibited differential expressions in the mutants (Figs. S7A2, A3), suggesting apoptosis pathways were activated in the mutants. Increased transcriptional levels of apoptosis inducing factors, such as *p53*, *baxa*, *caspase8*, and etc., showed up-regulated at 24 hpf and 48 hpf in the whole mutants (Fig. S7B). Also, protein levels of apoptosis inducers P53 and cleaved Casepase3 were increased obviously while apoptosis suppressor Bcl-2 decreased in the head in the mutants (Fig. 7A1 − A4). Besides, more Caspase3 immunofluorescence signals displayed in the retinal cells in *stmn4*^*−/−*^ at 48 hpf and 96 hpf (Fig. 7B1 − B9, S7C). These results suggested that retinal cells underwent apoptosis in the *stmn4*^*−/−*^ mutants.

**Fig. 8:**
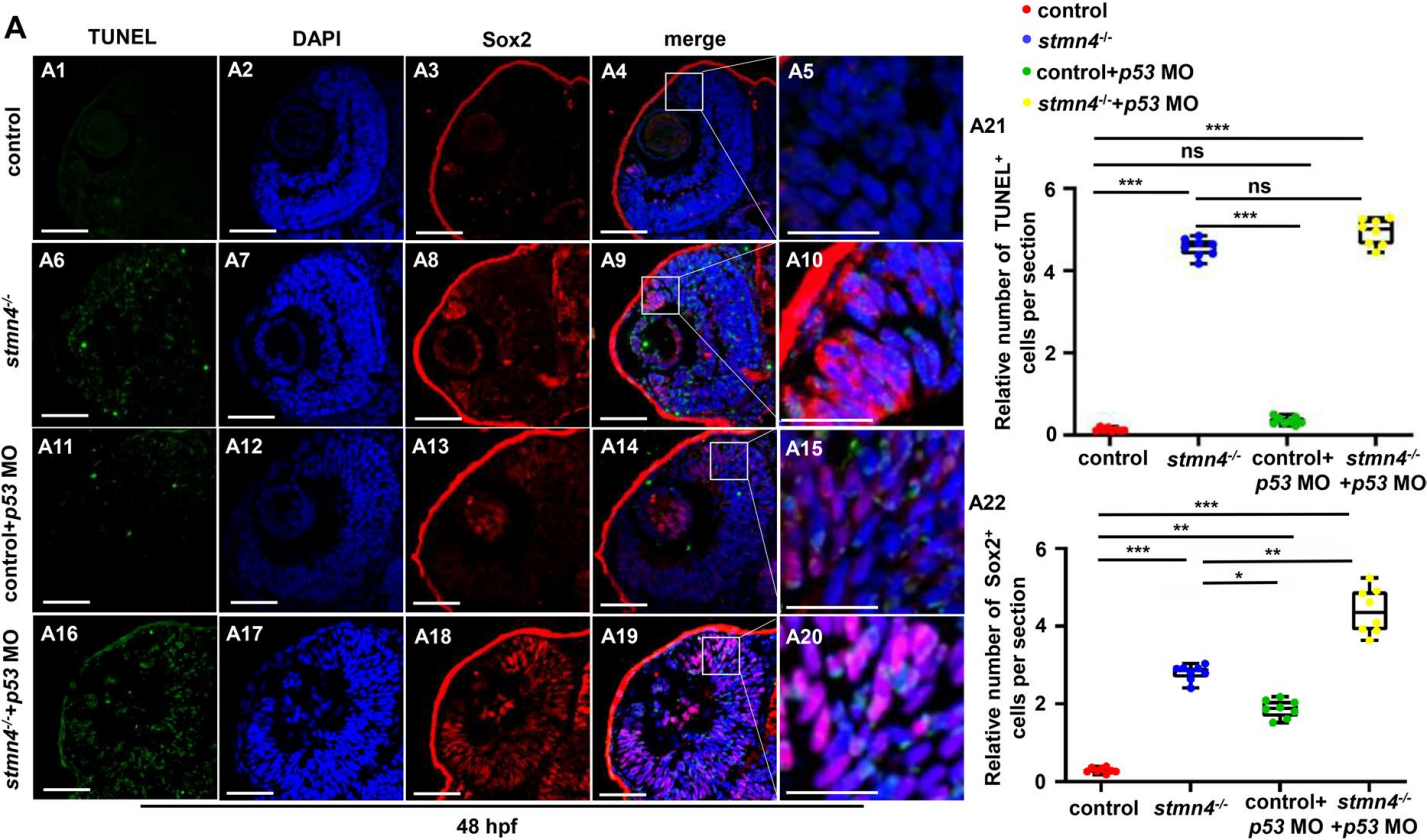
*Stmn4* deficiency induced apoptosis of RPCs independent on P53. (**A**) TUNEL and Sox2 staining in WT (A1 − A5), in *stmn4*^*−/−*^ (A6 − A10), in WT embryos injected with *p53* MO (A11 − A15), and in stmn4-/- injected with *p53* MO (A16 − A20) and the calculation data (A21 − A22). Scale bar, 50 μm (A1 − A4, A6 − A9, A11 − A14, A16 − A19), 25 μm (A5, A10, A15, A20). ***P < 0.001, **P < 0.01, *P < 0.05, ns, not significant

Next, we wonder whether the P53 pathway was mainly involved in the apoptosis of retinal cells in *stmn4*^*−/−*^. Thus, we injected *p53* morpholino (MO) (which will block the translation of *p53* transcripts) in zebrafish embryos (Li et al. 2023; Robu et al. 2007), to block P53 signaling in the *stmn4*^*−/−*^ mutants and the corresponding WT zebrafish, respectively. The TUNEL GFP positive signals could be observed in red Sox^2+^ cells in retina of *stmn4*^*−/−*^ mutants (Fig. 8A10) and the mutants co-injected with *p53* MO (Fig. 8A20), and more TUNEL positive signals were observed in the *p53* MO co-injected mutants (Fig. 8A1 − A22), not only suggesting that RPC Sox2^+^ cells underwent apoptosis, but also suggest that knockdown of *p53* in *stmn4*^*−/−*^ zebrafish further deteriorated the impaired differentiation of RPCs and led to the more accumulation of RPCs, and *p53* MO couldn’t rescue the apoptosis of retinal cells in the mutants. Together, these results suggested that activation of P53 pathway might be a compensatory mechanism for the impaired cell cycle and RPCs accumulation occurred in retina in the mutants, which might be not responsible for the occurred apoptosis.

## Discussion

Recently, we have reported that Stathmin family genes, especially *stmn4*, differentially expresses and exhibits down-regulated expressions in copper overload zebrafish embryos and HSPCs (Li et al. 2023). It is suggested that *stmn4* is closely related to the regulation of embryonic development and *Stathmins* are required for neural cell development and retinal regeneration (Beilharz et al. 1998; Levy et al. 2011). Cu overload induces retinal developmental defects in zebrafish embryos and larvae via triggering cell apoptosis (Zhao et al. 2020). Thus, in this study, we wonder whether the down-regulated expression of *stmn4* mediates Cu overload induces retinal developmental defects. Here, we unveil the reduced expression of *stmn4* in retina and that ectopic expression of *stmn4* mRNA could effectively rescue retinal developmental defects and the cell apoptosis in Cu overload embryos and larvae. Meanwhile, functional deficiency of *stmn4* induces impaired cell cycle in retinal cells, and leads to the accumulation of RPCs, which are also observed in Cu overload embryos and larvae, further suggesting the down-regulated expression of *stmn4* in retina of Cu overload embryos and larvae might be another potential contributor to their retinal developmental defects.

Cu has been unveiled to be spatial proximity to F-actin, especially at the basis of dendritic protrusions, suggesting that Cu might potentially modulate microtubule morphology in dendrites and spines (Domart et al. 2020). Additionally, the microtubule remodeling in response to Cu elevation has been directly demonstrated in the bone marrow mesenchymal stem cells (Chen et al. 2020). Recently, we unveil that Cu overload down-regulates expressions of microtubule genes and damages cytoskeleton morphology, then to lead to the impaired cell cycle and proliferation of hematopoietic stem and progenitor cells (HSPCs) during fish embryogenesis (Li et al. 2023). Normal expression of microtubule genes preserves cell morphology, and orderly and accurately microtubule rearrangements help advance the cell cycle (Heng and Koh 2010; Nunes and Ferreira 2021), while disruption of their integrity will lead to cell cycle stagnation (Blajeski et al. 2002; Heng and Koh 2010). Studies have reported that retinal cells in zebrafish are abnormal due to the cell cycle, and failure of progenitor cells exiting the cell cycle will result in the accumulation of RPCs (Baye and Link 2007). In this study, we unveil that the down-regulating expression of microtubule gene *stmn4*, might be the other attributor in mediating Cu overload induced the accumulation of RPCs and the subsequent retinal cell apoptosis via regulating cell cycle.

In this study, we demonstrate the novel roles of *stmn4* in retinal cell development and in cell cycle process. *Stmn4*^*−/−*^ embryos and larvae exhibit touch response defects and developmental defects of retinal cells, and exhibit general differentiation impairments of neural cells, such as neurons, astrocytes, and oligodendrocytes, suggesting normal functional *stmn4* is required for general neural cell differentiation.

Mature neural cells are functional items to ensure normal behavioral expression in vertebrates, thus, retinal cell developmental defects and general neural cell differentiation defects might potentially contribute to the touch response defects in *stmn4*^*−/−*^, implying the pivotal roles of neural system in regulating fish behaviors (Portugues and Engert 2009). Meanwhile, *stmn2b* is up-regulated in the *stmn4*^*−/−*^ mutants. Studies have shown that *stmn2b* is mainly expressed in the anterior central nervous system (the forebrain region, retina, optic tectum and hindbrain) and cranial ganglia starting from 48 hpf in zebrafish (Burzynski et al. 2009), the up-regulated expression of gene *stmn2b* may compensate for the retinal developmental defects in *stmn4*^*−/−*^ mutants as genetic compensation response (GCR) reported recently (El-Brolosy et al. 2019; Ma et al. 2019). A little of Stmn4 protein is still detected in the mutants at 24 hpf, we speculate that *stmn4* is maternal factor and the maternal Stmn4 protein might exist in the mutants in this study as studies reported recently (Hu et al. 2016; Li et al. 2021; Song et al. 2023).

In this study, depletion of *stmn4* in zebrafish leads to disorders in spindle assembly and in cell cycle exit, as well as M-phase arrest in retina, and results in the further cell apoptosis and the reduced retinal cells in the *stmn4*^*−/−*^ mutants. Meanwhile, in this study, genes related to the regulation of microtubule dynamics exhibit differential expressions and are significantly enriched in *stmn4*^*−/−*^ mutants. Stathmins are required in facilitating the mitosis and cell cycle progress via acting as microtubule destabilizers (Charbaut et al. 2001). Stathmin families have the SLD-like domain, which allows the family proteins to have similar regulating functions in the cells to be involved in mitosis by participating in the polymerization of microtubules (Chauvin and Sobel 2015). *Stmn4* has been less studied compared with other family genes, but it has been reported to play a role in activity-induced neuronal plasticity and neuronal differentiation (Beilharz et al. 1998), and have been proofed to influence the cell cycle progress via regulating the G_2_/M phase to regulate midbrain development before 24 hpf (Lin and Lee 2016), which is consistent with our findings that *stmn4* deficiency causes cell cycle arrest in the M phase and induces neuronal cell development defects during zebrafish embryogenesis. Disruption of microtubules can induce cell cycle arrest in G_2_/M phase and the formation of abnormal mitotic spindles (Kaur et al. 2014), and the M-phase arrests in retinal cells in *stmn4*^*−/−*^ mutants are observed in this study, suggesting that the dynamics of polymerization and depolymerization of microtubules (Gardner et al. 2008; Grenningloh et al. 2004) are damaged in the cells, further demonstrating the essential roles of Stathmin in cell cycle process (Hanash et al. 1988; Luo et al. 1991) via participating in microtubule assembly and regulating the depolymerization dynamics of microtubules (Desai and Mitchison 1997; Wäsch and Engelbert 2005).

In this study, we find that the expressions of cell cycle functional proteins (Cdc25b, Ccnb1 and Cdk1) are significantly down-regulated in *stmn4*^−/−^ mutants, consisting with the report that Stmn4 can indirectly control the process of neuronal cells entering to the G2/M phase by regulating the expression of Cdc25a in zebrafish midbrain (Lin and Lee 2016). The normal expression of CDK1/CCNB1 is the basic condition for cell exit from mitosis (Wäsch and Engelbert 2005). The down-regulated expression of Cdk1/Ccnb1 protein, the increased expression of RPC markers while reduced expression of mature neuron and rod/core cell markers and the increased PH^3+^ cells in retina, are observed in *stmn4*^*−/−*^ mutants, suggesting that RPCs encounter difficulties in exiting mitosis, which lead to the arrest of RPCs in M-phase. Combined with the aforementioned detection of the expression level of Tubulin and the observations of the mitotic process of RPCs, we demonstrate that the severely affected expression and function of Tubulin would lead to the difficulty of spindle formation. Taken the above points together, we demonstrate that *stmn4* deficiency induces cell cycle impairments in retina via down-regulating key cell cycle regulators Cdc25a, Ccnb1 and Cdk1 and damaging microtubule assembly dynamics, which jointly contribute to the finally developmental defects of retina and the resulted in touch response defects in the mutants.

The outcome of cells with arrested cell cycle is cell death, and microtubule dysfunction could easily lead to cell cycle arrest and even apoptosis with activated apoptotic signals in the cells (Iuchi et al. 2009; Liu et al. 2019; Nagireddy et al. 2022). Studies have shown that cell cycle arrest of RPCs may easily lead to cell apoptosis (Baye and Link 2007; Li et al. 2019, 2021) and the long-term stagnation of the M-phase naturally leads to cell apoptosis (Mc Gee 2015; Vitale et al. 2011; Vitovcova et al. 2020). In this study, we observe the enrichment of apoptosis related GO terms and the increase of apoptosis positive regulators P53 and cleaved Caspase3 while the decrease of apoptosis negative regulator Bcl-2 in *stmn4*^*−/−*^ mutants, which are prone to responding to cell cycle arrest caused by defective microtubule expression and to checking DNA and chromosome assembly (Liu et al. 2019). The changed expressions of P53, Caspase3, and Bcl-2 suggest the activation of apoptosis signaling, which subsequently induce cell apoptosis in retina in the mutants. Additionally, we observe the most periphery distribution of Sox2^+^ RSPCs in retina in *stmn4*^*−/−*^ mutants at 48 hpf, and the TUNEL signaling are also observed in the similar domain in retina in the mutants. Meanwhile, the overlapping of Sox2^+^ and TUNEL^+^ signals has been observed in retinal cells in the mutants, demonstrating the M-phase arrested RPCs or RSPCs might undergo cell apoptosis.

In this study, we unveil that knockdown of *p53* in *stmn4*^*−/−*^ embryos induces more accumulation of RPCs, suggesting more cell cycle arrests in RPCs occur in the mutants with *p53* knockdown. *P53* is a check protein in DNA and chromosome damages in cell cycle (Speidel 2015). Therefore, these results suggested that activation of P53 pathway may be a compensatory mechanism for the damaged microtubule assembly induced impaired cell cycle in the mutants, which may be not responsible for the subsequently occurred retinal cell apoptosis.

## Conclusions

Overall, this study confirms that *stmn4* deficiency leads to retinal developmental defects via affecting cell cycle progression and differentiation of RPCs and the subsequent cell apoptosis during zebrafish embryogenesis. The working model is illustrated in Graphical Abstract for an intuitive understanding of how *stmn4* defects induces retinal developmental defects during zebrafish embryogenesis. However, based on our current observations, we have not yet answered whether Cu stress can inhibit differentiation of neural progenitor cells via down-regulating *stmn4* expression. At the same time, this study cannot rule out the possibility that other untested genes or signals might mediate *stmn4* deficiency induced retinal developmental defects.

## Supplementary Information

Below is the link to the electronic supplementary material.

Supplementary file1 (DOCX 2171 KB)

## Data Availability

All data generated or analyzed in this study are present in this published article and its supplementary information files.
