# Supplementary material for: Deficiency of copper responsive gene stmn4 induces retinal developmental defects
Source: Cell Biol Toxicol. 2024 Jan 22;40(1):2. doi: 10.1007/s10565-024-09847-8 (PMC10803583; doi:10.1007/s10565-024-09847-8)
Supplement: Supplementary file 1 — Supplementary file1 (DOCX 2171 KB) [file 10565_2024_9847_MOESM1_ESM.docx]

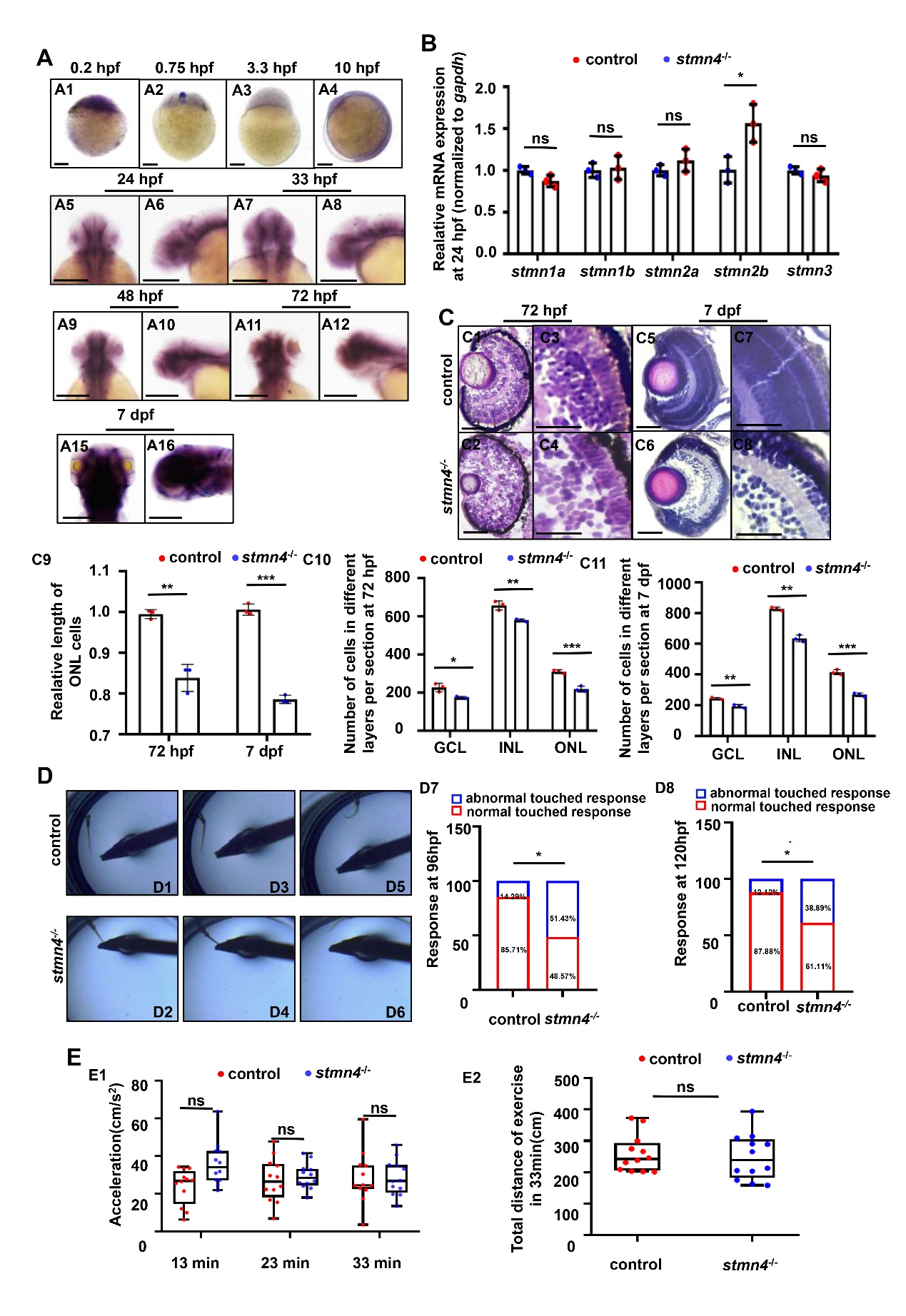


**Figure S1 *Stmn4* deficiency induced abnormal tissue and touch response in zebrafish embryos and larvae.** (**A**) *Stmn4* transcript distribution during zebrafish embryogenesis, mostly distributed in head and retinal cells (A1−A16). (**B**) The mRNA expression of other genes of Stathmin family in *stmn4^-/-^* mutants. (**C**) HE staining showed the difference in the eyes of WT and *stmn4*^-/-^embryos and larvae at 72 hpf and 7 dpf (C1−C8), and the calculation data (C9−C11). (**D**) Touch response assays in WT and *stmn4^-^*^/-^ larvae at 96 hpf (D1−D8). (**E**) Behavioral assays in WT and *stmn4^-^*^/-^ larvae at 96 hpf (E1, E2). A1−A4, A6, A8, A10, A12, A16, lateral view, anterior to the left, and dorsal to the up; A5, A7, A9, A11, A15, head to the up, and dorsal to the down. Scale bar, 200 μm (A1−A16), 100 μm (C1, C2, C5, C6), 50 μm (C3, C4, C7, C8). ****P*<0.001, ***P*<0.01, **P*<0.05, ns, not significant.


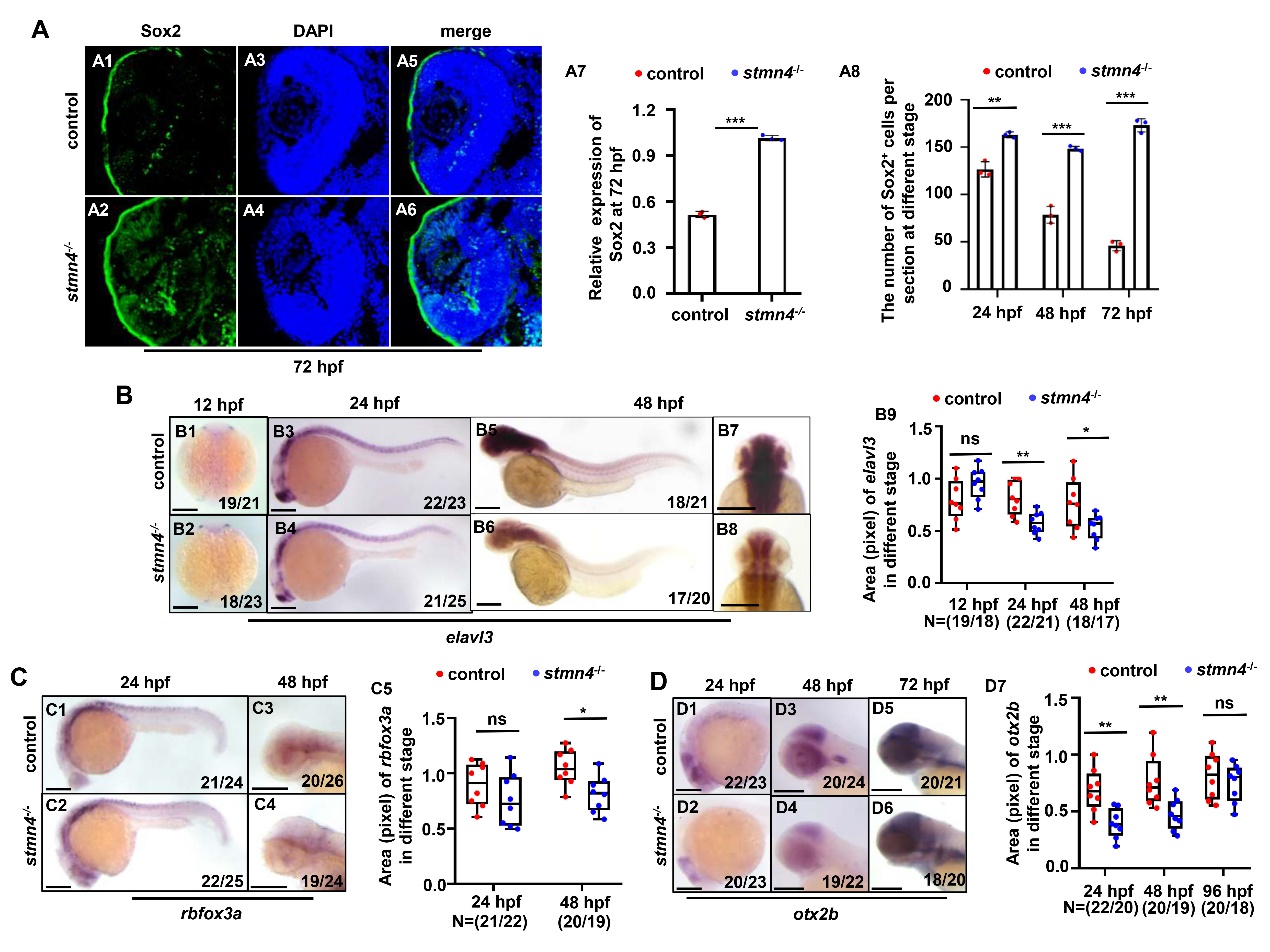


**Figure S2 *Stmn4* deletion led to neuronal progenitor cell block and neuronal differentiation deficiency.** (**A**) Immunofluorescence assays for the expression of neural progenitor marker Sox2 at 72 hpf in WT and *stmn4*^-/-^ embryos (A1−A6), and the calculation data (A7, A8). (B) The expression of neuronal marker elavl3 in WT and *stmn4^-/-^* mutated embryos at 12 hpf, 24 hpf, and 48 hpf (B1−B8), respectively, and the calculation of the relative expression levels (B9). (**C**) The expression of mature neuron marker *rbfox3a* in WT and *stmn4^-/-^* mutated embryos at 24 hpf and 48 hpf (C1−C4), respectively, and the calculation of the relative expression levels (C5). (**D**) The expression of *otx2b* in WT and *stmn4^-/-^* mutated embryos at 24 hpf, 48 hpf and 72 hpf (D1−D6), respectively, and the calculation of the relative expression levels (D7). B1−B6, C1−C4, D1−D6, lateral view, anterior to the left, and dorsal to the up; B7, B8, head to the up, and dorsal to the down. Scale bar, 200 μm (B1−B8, C1−C4, D1−D6). 50 μm (A1−A6). ****P*<0.001, ***P*<0.01, **P*<0.05, ns, not significant.


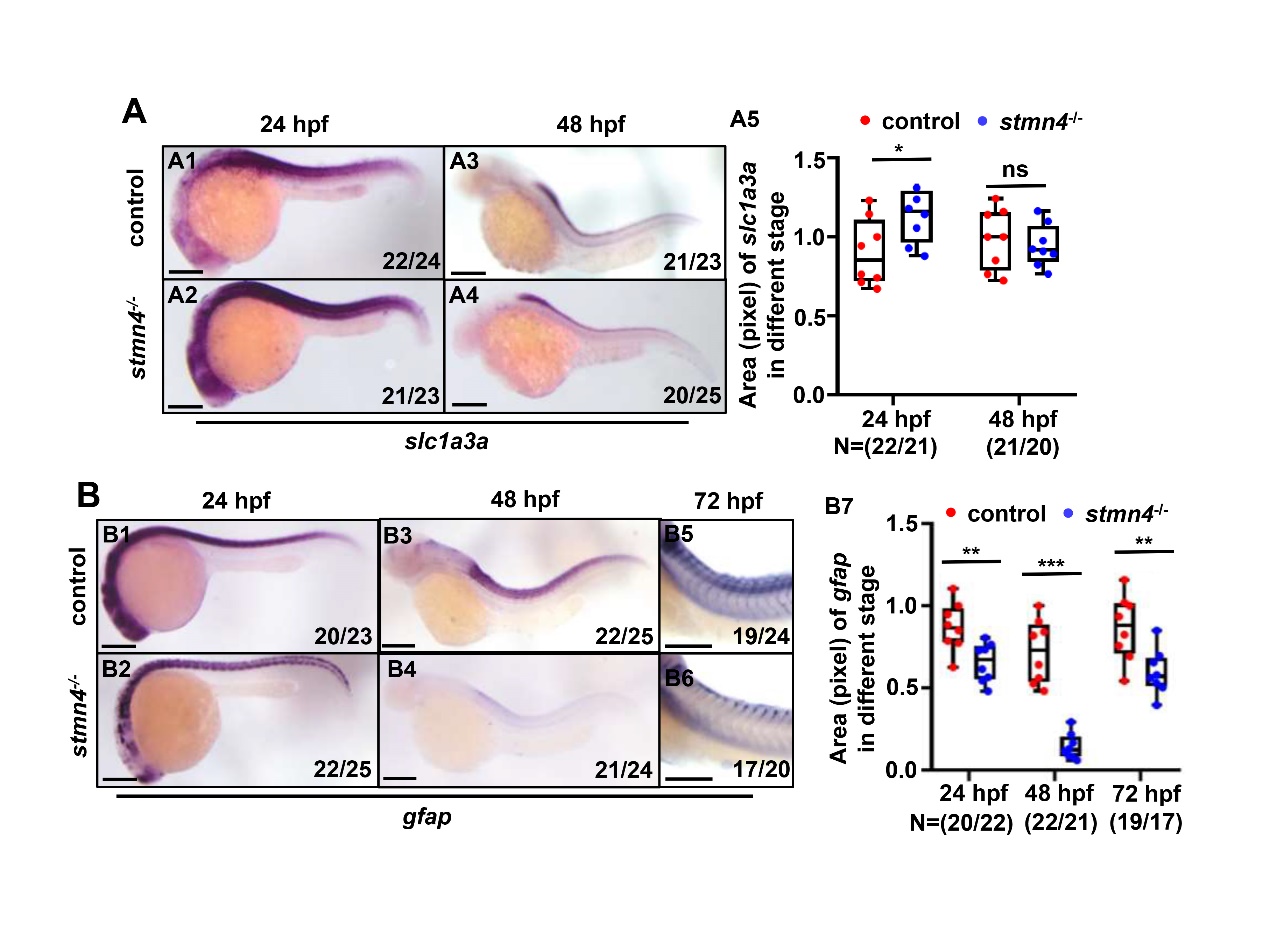


**Figure S3 *Stmn4* deficiency led to astrocyte differentiation defects.** (**A**) The expression of astrocyte progenitor marker *slc1a3a* in WT and *stmn4^-/-^* mutated embryos at 24 hpf and 48 hpf (A1−A4), respectively, and the calculation of the relative expression levels (A5). (**B**) The expression of mature astrocyte marker *gfap* in WT and *stmn4^-/-^* mutated embryos at 24 hpf, 48 hpf and 72 hpf (B1−B6), respectively, and the calculation of the relative expression levels (B7). A1−A4, B1−B6, lateral view, anterior to the left, and dorsal to the up. Scale bar, 200 μm (A1−A4, B1−B6). ****P*<0.001, ***P*<0.01, **P*<0.05, ns, not significant.


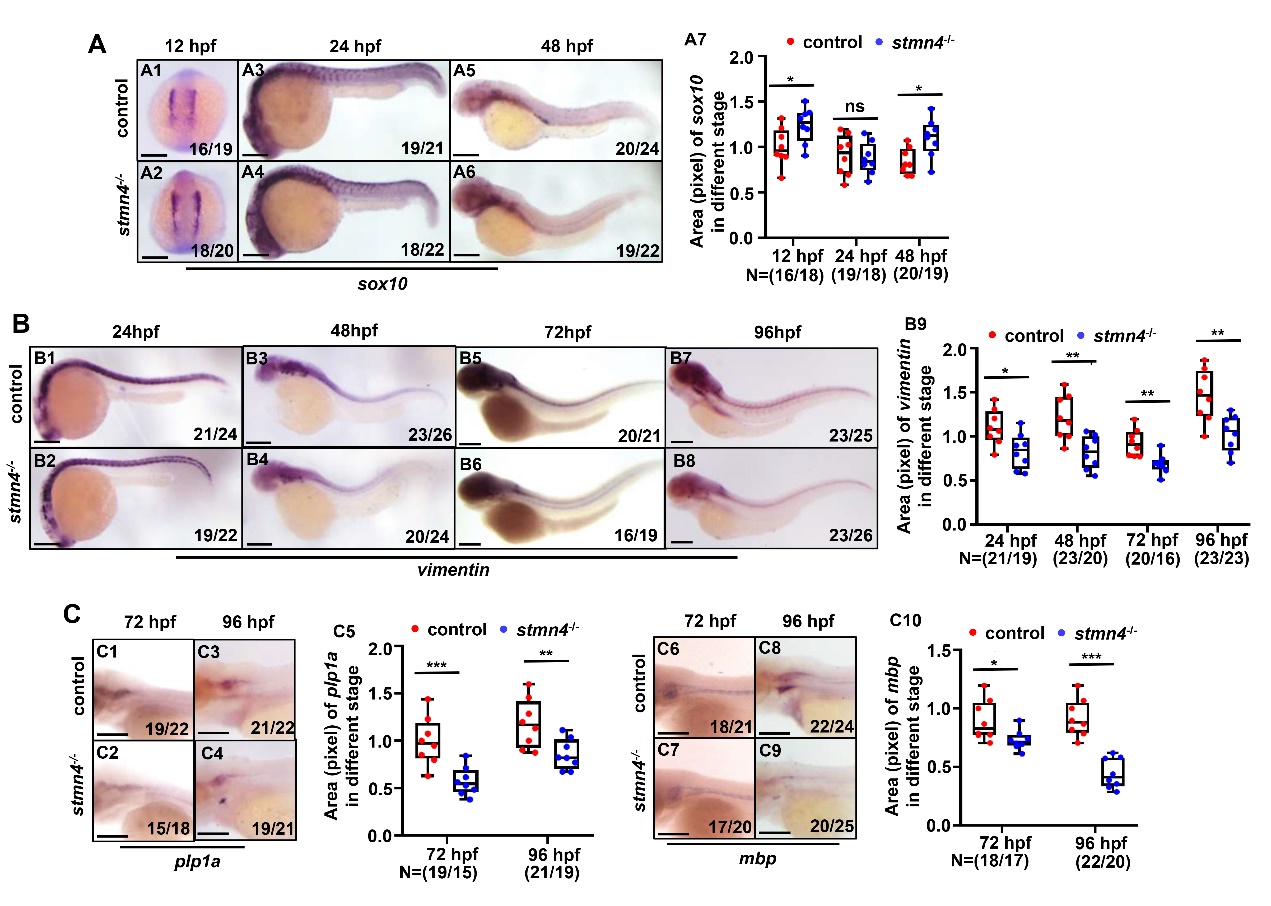


**Figure S4 *Stmn4* deficiency led to oligodendrocyte differentiation defects.** (**A**) The expression of oligodendrocyte progenitor marker *sox10* in WT and *stmn4^-/-^* mutated embryos at 12 hpf, 24 hpf and 48 hpf (A1−A6), respectively, and the calculation of the relative expression levels (A7). (**B, C**) The expression of mature oligodendrocyte markers *vimentin*, *plp1a*, and *mbp* in WT and *stmn4^-/-^* mutated embryos and larvae at 24 hpf, 48 hpf, 72 hpf and 96 hpf (B1−B8, C1−C4, C6−C9), respectively, and the calculation of the relative expression levels (B9, C5, C10). A1−A6, B1−B8, C1−C4, C6−C9, lateral view, anterior to the left, and dorsal to the up. Scale bar, 200 μm (A1−A6, B1−B8, C1−C4, C6−C9). ****P*<0.001, ***P*<0.01, **P*<0.05, ns, not significant.


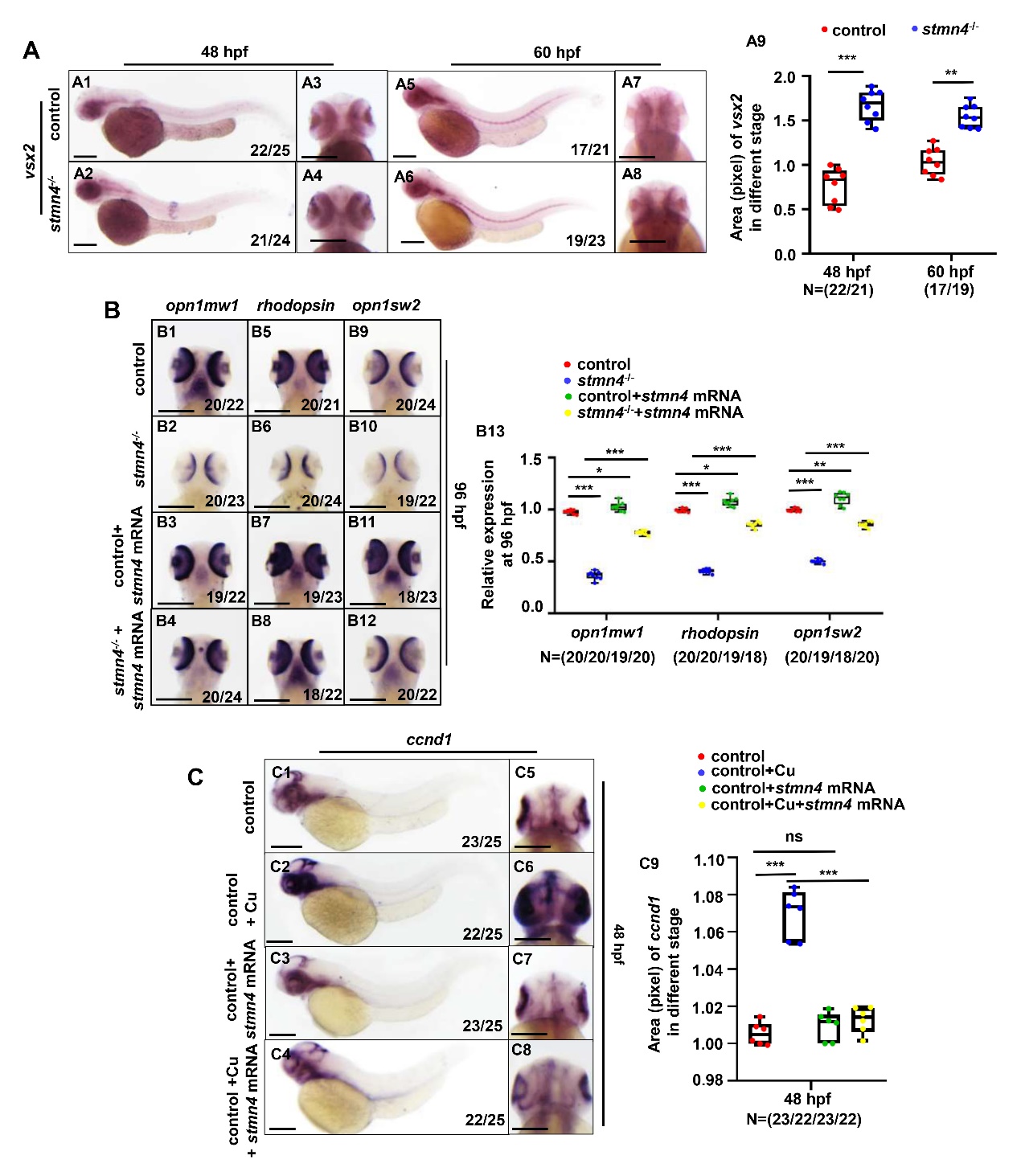


**Figure S5 *Stmn4* deficiency** **led to retinal progenitor cells differentiation defects.** (**A**) Expressions of neuron progenitor marker *vsx2* in retina in WT and *stmn4^-/-^* mutated embryos at 48 hpf and 60 hpf (A1−A8), respectively, and the calculation of the relative expression levels (A9). (**B**) *Stmn4* mRNA effectively rescued the expression of retinal *opn1mw1*, *rhodopsin*, and *opn1sw2* to nearly normal level at 96 hpf in *stmn4*^-/-^ zebrafish larvae (B1−B12), and the calculation of the relative expression levels (B13). (**C**) *Stmn4* mRNA effectively rescued the increased expression of *ccnd1* to nearly normal level in Cu stressed embryos (C1−C8), and the calculation of the relative expression levels (C9). A1, A2, A5, A6, C1−C4, lateral view, anterior to the left, and dorsal to the up; A3, A4, A7, A8, B1−B12, C5−C8, head to the up, and dorsal to the down. Scale bar, 200 μm (A1−A8, B1−B12, C1−C8). ****P*<0.001, ***P*<0.01, **P*<0.05, ns, not significant.


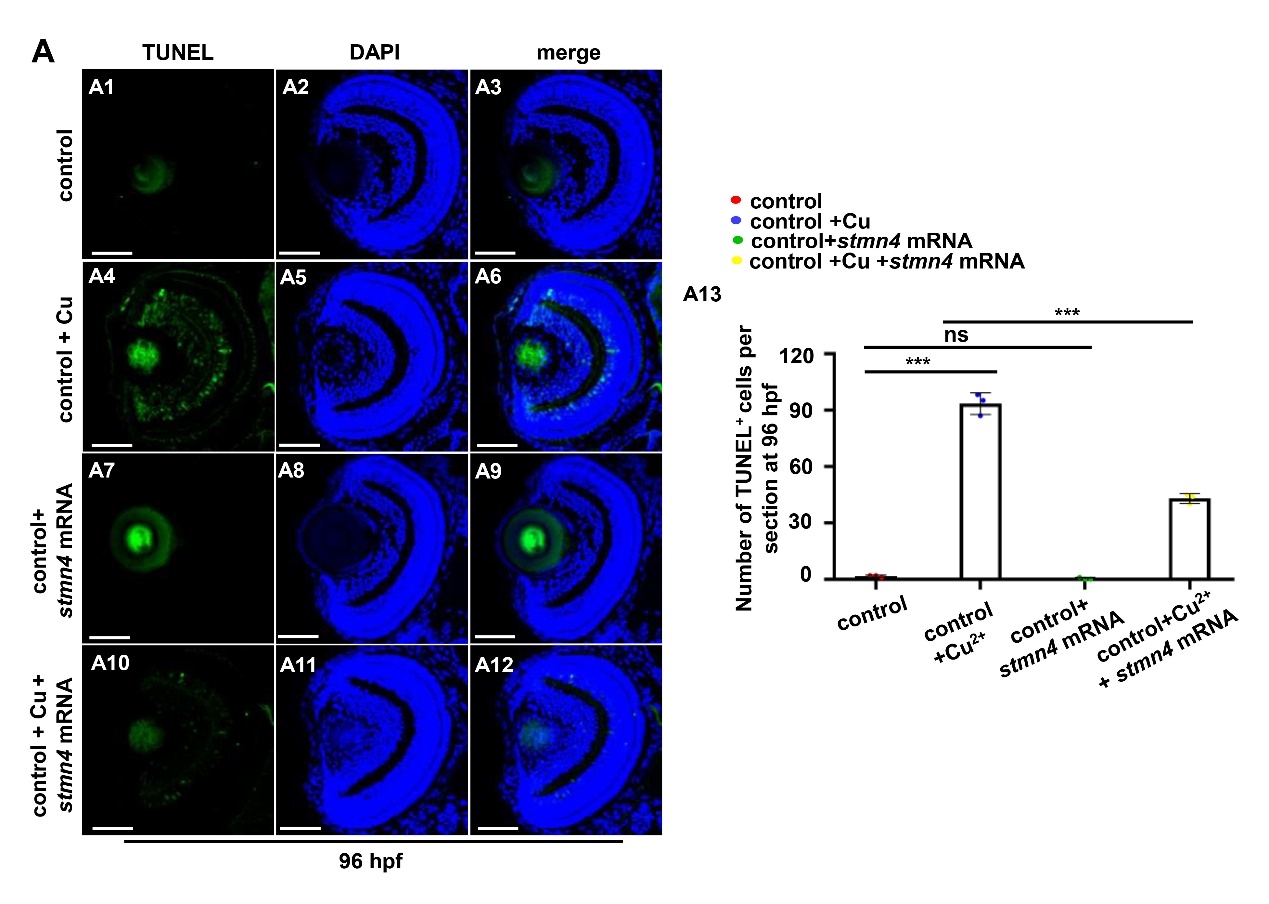


**Figure S6 *Stmn4* mRNA can effectively rescue apoptosis of retinal cells in copper stressed embryos.** TUNEL assays at 96 hpf in WT group, WT+ Cu group, WT+ *stmn4* mRNA group, WT+ Cu+ *stmn4* mRNA group zebrafish embryos (A1−A12) and the calculation data (A13). Scale bar, 50 μm (A1−A12). ****P*<0.001, ns, not significant.


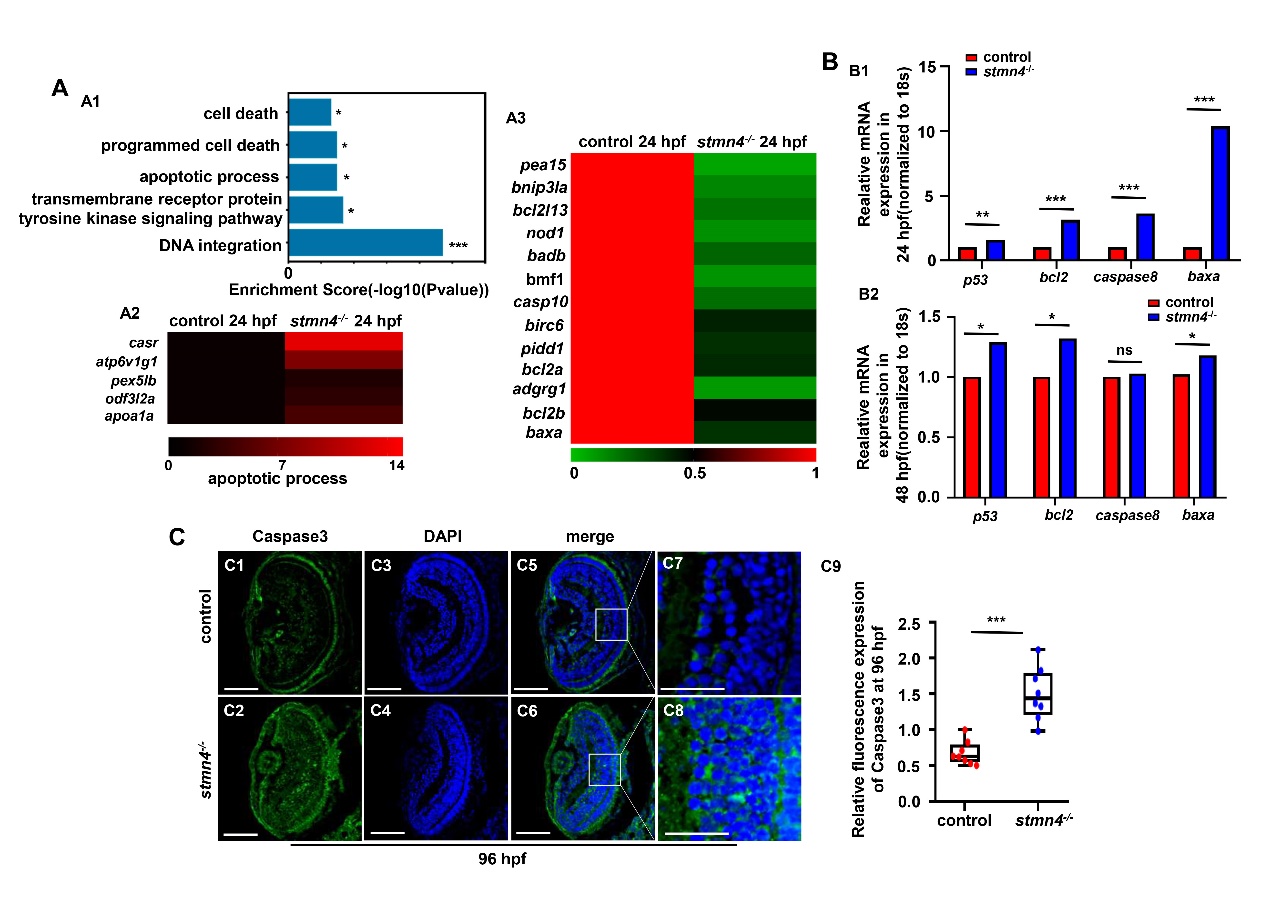


**Figure S7 *Stmn4* deficiency led to apoptosis of retinal cells in zebrafish embryos.** (**A**) Apoptosis-related GOs were enriched for DEGs in *stmn4*^-/-^ mutants at 24 hpf (A1). Heat maps for the apoptosis related DEGs at 24 hpf in WT and *stmn4*^-/-^ embryos (A2, A3). (**B**) qRT-PCR assays of apoptosis-related genes at 24 hpf and 48 hpf in WT and *stmn4*^-/-^ embryos (B1, B2). (**C**) Immunofluorescence assays of Caspase3 in WT and *stmn4*^-/-^ retina at 96 hpf (C1−C8), and the calculation of the relative expression levels (C9). Scale bar, 50 μm (C1−C6), 25 μm (C7, C8). ****P*<0.001, ***P*<0.01, **P*<0.05, ns, not significant.

| Supplementary Table 1 | | |
| --- | --- | --- |
| Gene | Full names of gene | Sequence （in 5’ to 3’ direction） |
| *18s*-F |  | CGAGCAGGAGATGGGAACC |
| *18s*-R |  | CAACGGAAACGCTCATTGC |
| *gapdh*-F | *glyceraldehyde-3-phosph ate dehydrogenase* | TCGCTAGTTGGCATCGTTTATG |
| *gapdh*-R |  | TCGCTAGTTGGCATCGTTTATG |
| *cyclina1*-F | *cyclin A1 (ccna1)* | CACATGGAGGATGGAAAACTG |
| *cyclina1*-R |  | CCTTCTCAATGCTGAGGAAGAC |
| *cyclina2*-F | *cyclin A2 (ccna2)* | GCTCCTCTTTCCAGTCGT |
| *cyclina2*-R |  | CTGCTCATTCTCGGTCAA |
| *cyclinb*-F | *cyclin B (ccnb1)* | TCGCCACCATTGACCTTC |
| *cyclinb*-R |  | GACTTGACCTCCATTTCC |
| *cyclind1*-F | *cyclin D1 (ccnd1)* | TGTTGAATGACCGAGTTT |
| *cyclind1*-R |  | TCCAGGTAGTTCATAGCC |
| *cycline1*-F | *cyclin E1 (ccne1)* | AACAACCTGCTCGGAAAA |
| *cycline1*-R |  | CCATGAAGCGATCAAAGTA |
| *cycling2*-F | *cyclin G2 (ccng2)* | TTGTCGCTGTTGATGCTT |
| *cycling2*-R |  | CGGTTTGGCACATTCTGG |
| *cenpf*-F | *centromere protein F* | CAGTCGGTTTCTAAGTGTTC |
| *cenpf*-R |  | CCTTGATTTTCTGCTCCA |
| *atm*-F | *ATM serine/threonine kinase* | TCAGCCTCCACTCAAGCA |
| *atm*-R |  | TGAAGAACCTCCACCACA |
| *p130*-F | *Retinoblastoma-like 2 (rbl2)* | ACATTGGGGAAAACTAAA |
| *p130*-R |  | CGACACGCAACATACAGAG |
| *cdc25b*-F | *cell division cycle 25B* | TTTTGCCTGCCGACCGAA |
| *cdc25b*-R |  | CCCTCCAGAAAGCCATCA |
| *p53*-F | *tumor protein p53* | TTGCCGGGATCGTTTGACC |
| *p53*-R |  | ATAGATGGCAGTGGCTCGAA |
| *baxa*-F | *bcl-2-Associated X* | CCGCCGCCCAATGAAATCTCCA |
| *baxa*-R |  | TTAAAGAGAACTGCAGATC |
| *bcl2a*-F | *bcl-2 apoptosis regulator a* | GACGGAGTGAACTGGGGGCGGAT |
| *bcl2a*-R |  | CCAAGCCCAGCGCCGCCAAGC |
| *caspase8*-F | *apoptosis-related cysteine peptidase* | TTAAGAAACTGAAAAGCAACT |
| *caspase8*-R |  | AACGCCTTTTGGGAATGAA |
| *stmn1a*-F | *stathmin 1a* | AAGGACCTTTCCTTGGTGGA |
| *stmn1a*-R |  | CATGCTCACGCTTTTCTGCTA |
| *stmn1b*-F | *stathmin 1b* | AGAAACAAAAGAGGGAGGCG |
| *stmn1b*-R |  | ATCCAAACACGGAAAACTGG |
| *stmn2a*-F | *stathmin 2a* | CGCTTACGTTTGCGGTGTT |
| *stmn2a*-R |  | TTTGTTCAGGAAAAGGTGGC |
| *stmn2b*-F | *stathmin 2b* | CCGGATGGCAGAGGAAAA |
| *stmn2b*-R |  | CCGGATGGCAGAGGAAAA |
| *stmn3*-F | *stathmin 3* | AAGGAGATTCACGCCGAAGA |
| *stmn3*-R |  | AAACTGCTGCCCAACTGCT |
| *stmn4*-F | *stathmin 4* | TGCGGTGGATCTCAACTGG |
| *stmn4*-R |  | GGGTCTTTACGCTGGGGAAT |

| Supplementary Table 2 | | |
| --- | --- | --- |
| Gene | Full names of gene | Sequence （in 5’ to 3’ direction） |
| *opn1lw1*-probe-F | *opsin 1（cone pigments），long-wave-sensitive, 1* | TGAGGGTCCCAATTACCA |
| *opn1lw1*-probe-R |  | TAATACGACTCACTATAGGGA  GCAGATGCCCATTTAGC |
| *opn1sw2*-probe-F | *opsin 1 （cone pigments）short-wave-sensitive 2* | CCTCGGGAACTTTACCTT |
| *opn1sw2*-probe-R |  | TAATACGACTCACTATAG  GGTTGGAAACCACCCAGATT |
| *rhodopsin*-probe-F | *rhodopsin* | AACCTGGAGGGCTTCTTT |
| *rhodopsin*-probe-R |  | TAATACGACTCACTATAGG  GTATGACTAACAGTGGGATGA |
| *opn1mw1*-probe-F | *opsin 1 (cone pigments), medium-wave-sensitive, 1* | GCATACCCGTAACCACAAT |
| *opn1mw1*-probe-R |  | TAATACGACTCACTATAGG  GGCAAACAAGAAGCCCAAA |
| *vsx2*-probe-F | *visual system homeobox 2* | GTCACTCAGACACCGACC |
| *vsx2*-probe-R |  | TAATACGACTCACTATAGGG  TGATCTATACATGGCTCAT |
| *ccnd1*-probe-F | *cyclin D1* | CAACTTCATCGCAAGCC |
| *ccnd1*-probe-R |  | TAATACGACTCACTATAGGGC  GGTCATCAAAGCCACA |
| *crx*-probe-F | *cone-rod homeobox protein* | CGTCGTTGGGCTTCAGTTC |
| *crx*-probe-R |  | TAATACGACTCACTATAGGGTCT  TCTTCACGCATCTTTCCTT |
| *elavl3*-probe-F | *elav like RNA binding protein 3* | GCGCACGTCCTACAACTACA |
| *elavl3*-probe-R |  | TAATACGACTCACTATAGGG  GCATCTGGATCTGCATCTCA |
| *sox2*-probe-F | *sex determining region Y-box（SRY） transcription factor 2* | CTGTCCGAGAGCGAGAAGC |
| *sox2*-probe-R |  | TAATACGACTCACTATAGGG  GGAATGAGACGACGACGTGA |
| *gfap*-probe-F | *glial fibrillary acidic protein* | CCTCAGACCTCGGCACTCTC |
| *gfap*-probe-R |  | TAATACGACTCACTATAGGG  GCCATCTCCTCCTTCAGCAT |
| *sox10-*probe-F | *SRY-box transcription factor 10* | GCGAGGCGGTCAGTCAGGT |
| *sox10-*probe-R |  | TAATACGACTCACTATAGGGGT  GGTAGGGGGCGTTGGAG |
| *otx2b-*probe-F | *orthodenticle homeobox 2b* | CTCTGTCCAACCAACACCCTAA |
| *otx2b-*probe-R |  | TAATACGACTCACTATAGGGGAG  AAAGCCTGCCCATTCC |
| *slc1a3a-*probe-F | *solute carrier family 1 member 3a* | AATAACGGCACGCAGGAGC |
| *slc1a3a-*probe-R |  | TAATACGACTCACTATAGGGA  TGGGCAGAGTGGCAGAAC |
